# Proximal Distance Algorithms: Theory and Practice

**Published:** 2019-04

**Authors:** Kevin L. Keys, Hua Zhou, Kenneth Lange

**Affiliations:** Department of Medicine, University of California, San Francisco, CA 94158, USA; Department of Biostatistics, University of California, Los Angeles, CA 90095-1772, USA; Departments of Biomathematics, Human Genetics, and Statistics, University of California, Los Angeles, CA 90095-1766, USA

**Keywords:** constrained optimization, EM algorithm, majorization, projection, proximal operator

## Abstract

Proximal distance algorithms combine the classical penalty method of constrained minimization with distance majorization. If *f*(*x*) is the loss function, and *C* is the constraint set in a constrained minimization problem, then the proximal distance principle mandates minimizing the penalized loss $f(x)+\frac{\rho }{2}\text{dist}{\left(x,C\right)}^{2}$ and following the solution *x*_*ρ*_ to its limit as *ρ* tends to ∞. At each iteration the squared Euclidean distance dist(*x,C*)^2^ is majorized by the spherical quadratic ‖*x*− *P*_*C*_(*x*_*k*_)‖^2^, where *P*_*C*_(*x*_*k*_) denotes the projection of the current iterate *x*_*k*_ onto *C*. The minimum of the surrogate function $f(x)+\frac{\rho }{2}\|x-P_{C}\left(x_{k}\right){\|}^{2}$ is given by the proximal map prox_*ρ*_−_1*f*_[*P*_*C*_(*x*_*k*_)]. The next iterate *x*_*k*+1_ automatically decreases the original penalized loss for fixed *ρ*. Since many explicit projections and proximal maps are known, it is straightforward to derive and implement novel optimization algorithms in this setting. These algorithms can take hundreds if not thousands of iterations to converge, but the simple nature of each iteration makes proximal distance algorithms competitive with traditional algorithms. For convex problems, proximal distance algorithms reduce to proximal gradient algorithms and therefore enjoy well understood convergence properties. For nonconvex problems, one can attack convergence by invoking Zangwill’s theorem. Our numerical examples demonstrate the utility of proximal distance algorithms in various high-dimensional settings, including a) linear programming, b) constrained least squares, c) projection to the closest kinship matrix, d) projection onto a second-order cone constraint, e) calculation of Horn’s copositive matrix index, f) linear complementarity programming, and g) sparse principal components analysis. The proximal distance algorithm in each case is competitive or superior in speed to traditional methods such as the interior point method and the alternating direction method of multipliers (ADMM). Source code for the numerical examples can be found at https://github.com/klkeys/proxdist.

## 1. Introduction

The solution of constrained optimization problems is part science and part art. As mathematical scientists explore the largely uncharted territory of high-dimensional nonconvex problems, it is imperative to consider new methods. The current paper studies a class of optimization algorithms that combine Courant’s penalty method of optimization (Beltrami, 1970; Courant, 1943) with the notion of a proximal operator (Bauschke and Combettes, 2011; Moreau, 1962; Parikh and Boyd, 2013). The classical penalty method turns constrained minimization of a function *f*(*x*) over a closed set *C* into unconstrained minimization. The general idea is to seek the minimum point of a penalized version *f*(*x*)+*ρq*(*x*) of *f*(*x*), where the penalty *q*(*x*) is nonnegative and vanishes precisely on *C*. If one follows the solution vector *x*_*ρ*_ as *ρ* tends to ∞, then in the limit one recovers the constrained solution. The penalties of choice in the current paper are squared Euclidean distances dist(*x,C*)^2^ = inf_*y*∈*C*_ ‖*x*−*y*‖^2^.

The formula

(1) $${\text{prox}}_{f}\left(y\right)={\text{argmin}}_{x}\left[f\left(x\right)+\frac{1}{2}{\left\|x-y\right\|}^{2}\right]$$

defines the proximal map of a function *f*(***x***). Here ‖ · ‖ is again the standard Euclidean norm, and *f*(***x***) is typically assumed to be closed and convex. Projection onto a closed convex set *C* is realized by choosing *f*(***x***) to be the 0*/*∞ indicator *δ*_*C*_(***x***) of *C*. It is possible to drop the convexity assumption if *f*(***x***) is nonnegative or coercive. In so doing, prox_*f*_(*y*) may become multi-valued. For example, the minimum distance from a nonconvex set to an exterior point may be attained at multiple boundary points. The point ***x*** in the definition (1) can be restricted to a subset *S* of Euclidean space by replacing *f*(***x***) by *f*(***x***) + *δ*_*S*_(***x***), where *δ*_*S*_(***x***) is the indicator of *S*.

One of the virtues of exploiting proximal operators is that they have been thoroughly investigated. For a large number of functions *f*(***x***), the map prox_*cf*_(***y***) for *c >* 0 is either given by an exact formula or calculable by an efficient algorithm. The known formulas tend to be highly accurate. This is a plus because the classical penalty method suffers from ill conditioning for large values of the penalty constant. Although the penalty method seldom delivers exquisitely accurate solutions, moderate accuracy suffices for many problems.

There are ample precedents in the optimization literature for the proximal distance principle. Proximal gradient algorithms have been employed for many years in many contexts, including projected Landweber, alternating projection onto the intersection of two or more closed convex sets, the alternating-direction method of multipliers (ADMM), and fast iterative shrinkage thresholding algorithms (FISTA) (Beck and Teboulle, 2009; Combettes and Pesquet, 2011; Landweber, 1951). Applications of distance majorization are more recent (Chi et al., 2014; Lange and Keys, 2014; Xu et al., 2017). The overall strategy consists of replacing the distance penalty dist(*x,C*)^2^ by the spherical quadratic ‖***x*** − ***y***_*k*_‖^2^, where ***y***_*k*_ is the projection of the *k*th iterate ***x***_*k*_ onto *C*. To form the next iterate, one then sets

$$x_{k+1}={\text{prox}}_{\rho^{-1}f}\left(y_{k}\right)\text{ with }y_{k}=P_{C}\left(x_{k}\right).$$

The MM (majorization-minimization) principle guarantees that *x*_*k*+1_ decreases the penalized loss. We call the combination of Courant’s penalty method with distance majorization the *proximal distance principle*. Algorithms constructed according to the principle are *proximal distance algorithms*.

The current paper extends and deepens our previous preliminary treatments of the proximal distance principle. Details of implementation such as Nesterov acceleration matter in performance. We have found that squared distance penalties tend to work better than exact penalties. In the presence of convexity, it is now clear that every proximal distance algorithm reduces to a proximal gradient algorithm. Hence, convergence analysis can appeal to a venerable body of convex theory. This does not imply that the proximal distance algorithm is limited to convex problems. In fact, its most important applications may well be to nonconvex problems. A major focus of this paper is on practical exploration of the proximal distance algorithm.

In addition to reviewing the literature, the current paper presents some fresh ideas. Among the innovations are: a) recasting proximal distance algorithms with convex losses as concave-convex programs, b) providing new perspectives on convergence for both convex and nonconvex proximal distance algorithms, c) demonstrating the virtue of folding constraints into the domain of the loss, and d) treating in detail seven interesting examples. It is noteworthy that some our new convergence theory is pertinent to more general MM algorithms.

It is our sincere hope to enlist other mathematical scientists in expanding and clarifying this promising line of research. The reviewers of the current paper have correctly pointed out that we do not rigorously justify our choices of the penalty constant sequence *ρ*_*k*_. The recent paper by Li et al. (2017) may be a logical place to start in filling this theoretical gap. They deal with the problem of minimizing *f*(***x***) subject to ***Ax*** = ***b*** through the quadratic penalized objective $f(x)+\frac{\rho }{2}\|Ax-b{\|}^{2}$. For the right choices of the penalty sequence *ρ*_*k*_, their proximal gradient algorithm achieves a *O*(*k*^−1^) rate of convergence for *f*(***x***) strongly convex. As a substitute, we explore the classical problem of determining how accurately the solution ***y***_*ρ*_ of the problem ${\text{min}}_{x}f(x)+\frac{\rho }{2}q{\left(x\right)}^{2}$ approximates the solution ***y*** of the constrained problem min_*x*∈*C*_ *f*(*x*). Polyak (1971) demonstrates that *f*(***y***)−*f*(***y***_*ρ*_) = *O*(***ρ***^−1^) for a penalty function *q*(***x***) that vanishes precisely on *C*. Polyak’s proof relies on strong differentiability assumptions. Our proof for the case *q*(***x***) = dist(***x****,C*) relies on convexity and is much simpler.

As a preview, let us outline the remainder of our paper. Section 2 briefly sketches the underlying MM principle. We then show how to construct proximal distance algorithms from the MM principle and distance majorization. The section concludes with the derivation of a few broad categories proximal distance algorithms. Section 3 covers convergence theory for convex problems, while Section 4 provides a more general treatment of convergence for nonconvex problems. To avoid breaking the flow of our exposition, all proofs are relegated to the Appendix. Section 5 discusses our numerical experiments on various convex and nonconvex problems. Section 6 closes by indicating some future research directions.

## 2. Derivation

The derivation of our proximal distance algorithms exploits the majorization-minimization (MM) principle (Hunter and Lange, 2004; Lange, 2010). In minimizing a function *f*(***x***), the MM principle exploits a surrogate function *g*(***x*** | ***x***_*k*_) that majorizes *f*(***x***) around the current iterate ***x***_*k*_. Majorization mandates both domination *g*(***x*** | ***x***_*k*_) ≥ *f*(***x***) for all feasible ***x*** and tangency *g*(***x***_*k*_ | ***x***_*k*_) = *f*(***x***_*k*_) at the anchor ***x***_*k*_. If ***x***_*k*+1_ minimizes *g*(***x*** | ***x***_*k*_), then the descent property *f*(***x***_*k*+1_) ≤ *f*(***x***_*k*_) follows from the string of inequalities and equalities

$$f\left(x_{k+1}\right)\leq g\left(x_{k+1}|x_{k}\right)\leq g\left(x_{k}|x_{k}\right)=f\left(x_{k}\right).$$

Clever selection of the surrogate *g*(***x*** | ***x***_*k*+1_) can lead to a simple algorithm with an explicit update that requires little computation per iterate. The number of iterations until convergence of an MM algorithm depends on how tightly *g*(***x*** | ***x***_*k*_) hugs *f*(***x***). Constraint satisfaction is built into any MM algorithm. If maximization of *f*(***x***) is desired, then the objective *f*(***x***) should dominate the surrogate *g*(***x*** | ***x***_*k*_) subject to the tangency condition. The next iterate ***x***_*k*+1_ is then chosen to maximize *g*(***x*** | ***x***_*k*_). The minorization-maximization version of the MM principle guarantees the ascent property.

The constraint set *C* over which the loss *f*(***x***) is minimized can usually be expressed as an intersection $\cap_{i=1}^{m}C_{i}$ of closed sets. It is natural to define the penalty

$$q\left(x\right)=\frac{1}{2}\overset{m}{\underset{i=1}{\sum }}\alpha_{i}\text{dist}{\left(x,C_{i}\right)}^{2}$$

using a convex combination of the squared distances. The neutral choice $\alpha_{i}=\frac{1}{m}$ is one we prefer in practice. Distance majorization gives the surrogate function

$$g_{\rho }\left(x|x_{k}\right)=f\left(x\right)+\frac{\rho }{2}\overset{m}{\underset{i=1}{\sum }}\alpha_{i}{\left\|x-P_{C_{i}}\left(x_{k}\right)\right\|}^{2}\;=f\left(x\right)+\frac{\rho }{2}{\left\|x-\overset{m}{\underset{i=1}{\sum }}\alpha_{i}P_{C_{i}}\left(x_{k}\right)\right\|}^{2}+c_{k}$$

for an irrelevant constant *c*_*k*_. If we put $y_{k}=\sum_{i=1}^{m}\alpha_{i}P_{C_{i}}\left(x_{k}\right)$, then by definition the minimum of the surrogate *g*_*ρ*_(*x* | *x*_*k*_) occurs at the proximal point

(2) $$x_{k+1}={\text{prox}}_{\rho^{-1}f}\left(y_{k}\right).$$

We call this MM algorithm the proximal distance algorithm. The penalty *q*(***x***) is generally smooth because

$$\nabla \frac{1}{2}\text{dist}{\left(x,C\right)}^{2}=x-P_{C}\left(x\right)$$

at any point ***x*** where the projection *P*_*C*_(***x***) is single valued (Borwein and Lewis, 2006; Lange, 2016). This is always true for convex sets and almost always true for nonconvex sets. For the moment, we will ignore the possibility that *P*_*C*_(***x***) is multi-valued.

For the special case of projection of an external point *z* onto the intersection *C* of the closed sets *C*_*i*_, one should take $f(x)=\frac{1}{2}\|z-x{\|}^{2}$. The proximal distance iterates then obey the explicit formula

$$x_{k+1}=\frac{1}{1+\rho }\left(z+\rho y_{k}\right).$$

Linear programming with arbitrary convex constraints is another example. Here the loss is *f*(***x***) = ***v***^*t*^***x***, and the update reduces to

$$x_{k+1}=y_{k}-\frac{1}{\rho }v.$$

If the proximal map is impossible to calculate, but *f*(***x***) is *L*-smooth (∇*f*(***x***) is Lipschitz with constant *L*), then one can substitute the standard majorization

$$f\left(x\right)\leq f\left(x_{k}\right)+\nabla f{\left(x_{k}\right)}^{t}\left(x-x_{k}\right)+\frac{L}{2}{\left\|x-x_{k}\right\|}^{2}$$

for *f*(***x***). Minimizing the sum of the loss majorization plus the penalty majorization leads to the MM update

(3) $$x_{k+1}=\frac{1}{L+\rho }\left[-\nabla f\left(x_{k}\right)+Lx_{k}+\rho y_{k}\right]\;=x_{k}-\frac{1}{L+\rho }\left[\nabla f\left(x_{k}\right)+\rho \nabla q\left(x_{k}\right)\right].$$

This is a gradient descent algorithm without an intervening proximal map.

In moderate-dimensional problems, local quadratic approximation of *f*(***x***) can lead to a viable algorithm. For instance, in generalized linear statistical models, Xu et al. (2017) suggest replacing the observed information matrix by the expected information matrix. The latter matrix has the advantage of being positive semidefinite. In our notation, if ***A***_*k*_ ≈ *d*^2^*f*(***x***_*k*_), then an approximate quadratic surrogate is

$$f\left(x_{k}\right)+\nabla f{\left(x_{k}\right)}^{t}\left(x-x_{k}\right)+\frac{1}{2}{\left(x-x_{k}\right)}^{t}A_{k}\left(x-x_{k}\right)+\frac{\rho }{2}{\left\|x-y_{k}\right\|}^{2}.$$

The natural impulse is to update *x* by the Newton step

(4) $$x_{k+1}=x_{k}-{\left(A_{k}+\rho I\right)}^{-1}\left[\nabla f\left(x_{k}\right)-\rho y_{k}\right].$$

This choice does not necessarily decrease *f*(***x***). Step halving or another form of backtracking restores the descent property.

A more valid concern is the effort expended in matrix inversion. If ***A***_*k*_ is dense and constant, then extracting the spectral decomposition ***VDV***^*t*^ of ***A*** reduces formula (4) to

$$x_{k+1}=x_{k}-V{\left(D+\rho I\right)}^{-1}V^{t}\left[\nabla f\left(x_{k}\right)-\rho y_{k}\right],$$

which can be implemented as a sequence of matrix-vector multiplications. Alternatively, one can take just a few terms of the series

$${\left(A_{k}+\rho I\right)}^{-1}=\rho^{-1}\overset{\infty }{\underset{j=0}{\sum }}{\left(-\rho^{-1}A_{k}\right)}^{j}$$

when *ρ* is sufficiently large. For a generalized linear model, parameter updating involves solving the linear system

(5) $$\left(Z^{t}W_{k}Z+\rho I\right)x=Z^{t}W_{k}^{1/2}v_{k}+\rho y_{k}$$

for ***W***_*k*_ a diagonal matrix with positive diagonal entries. This task is equivalent to minimizing the least squares criterion

(6) $${\left\|\left(\begin{array}{c} W_{k}^{1/2}Z\\ \sqrt{\rho }I \end{array}\right)x-\left(\begin{array}{c} v_{k}\\ \sqrt{\rho }y_{k} \end{array}\right)\right\|}^{2}.$$

In the unweighted case, extracting the singular value decomposition ***Z*** = ***USV***^*T*^ facilitates solving the system of equations (5). The svd decomposition is especially cheap if there is a substantial mismatch between the number rows and columns of ***Z***. For sparse ***Z***, the conjugate gradient algorithm adapted to least squares (Paige and Saunders, 1982b) is subject to much less ill conditioning than the standard conjugate gradient algorithm. Indeed, the algorithm LSQR and its sparse version LSMR (Fong and Saunders, 2011) perform well even when the matrix ${\left(Z^{t}W_{k}^{1/2},\sqrt{\rho }I\right)}^{t}$ is ill conditioned.

The proximal distance principle also applies to unconstrained problems. For example, consider the problem of minimizing a penalized loss ℓ(***x***)+*p*(***Ax***). The presence of the linear transformation ***Ax*** in the penalty complicates optimization. The strategy of parameter splitting introduces a new variable ***y*** and minimizes ℓ(***x***) + *p*(***y***) subject to the constraint ***y*** = ***Ax***. If *P*_*M*_(*z*) denotes projection onto the manifold

M={z=(x,y):Ax=y},

then the constrained problem can be solved approximately by minimizing the function

$$l\left(x\right)+p\left(y\right)+\frac{\rho }{2}\text{dist}{\left(z,M\right)}^{2}$$

for large *ρ*. If *P*_*M*_(*z*_*k*_) consists of two subvectors ***u***_*k*_ and ***v***_*k*_ corresponding to ***x***_*k*_ and ***y***_*k*_, then the proximal distance updates are

$$x_{k+1}={\text{prox}}_{\rho^{-1}l}\left(u_{k}\right)\text{ and }y_{k+1}={\text{prox}}_{\rho^{-1}p}\left(v_{k}\right).$$

Given the matrix ***A*** is *n* × *p*, one can attack the projection by minimizing the function

$$q\left(x\right)=\frac{1}{2}{\left\|x-u\right\|}^{2}+\frac{1}{2}{\left\|Ax-v\right\|}^{2}.$$

This leads to the solution

$$x={\left(I_{p}+A^{t}A\right)}^{-1}\left(A^{t}v+u\right)\text{ and }y=Ax.$$

If *n < p*, then the Woodbury formula

$${\left(I_{p}+A^{t}A\right)}^{-1}=I_{p}-A^{t}{\left(I_{n}+AA^{t}\right)}^{-1}A$$

reduces the expense of matrix inversion.

Traditionally, convex constraints have been posed as inequalities *C* = {***x*** : *a*(***x***) ≤ *t*}. Parikh and Boyd (2013) point out how to project onto such sets. The relevant Lagrangian for projecting an external point ***y*** amounts to

$$L\left(x,\lambda \right)=\frac{1}{2}{\left\|y-x\right\|}^{2}+\lambda \left[a\left(x\right)-t\right]$$

with *λ* ≥ 0. The corresponding stationarity condition

(7) 0=x−y+λ∇a(x),

can be interpreted as *a*[prox_*λa*_(***y***)] = *t*. One can solve this one-dimensional equation for *λ* by bisection. Once *λ* is available, ***x*** = prox_*λa*_(***y***) is available as well. Parikh and Boyd (2013) note that the value *a*[prox_*λa*_(***y***)] is decreasing in *λ*. One can verify their claim by implicit differentiation of equation (7). This gives

$$\frac{d}{d\lambda }x=-{\left[I+\lambda d^{2}a\left(x\right)\right]}^{-1}\nabla a\left(x\right)$$

and consequently the chain rule inequality

$$\frac{d}{d\lambda }a\left[{\text{prox}}_{\lambda a}\left(y\right)\right]=-da\left(x\right){\left[I+\lambda d^{2}a\left(x\right)\right]}^{-1}\nabla a\left(x\right)\leq 0.$$

## 3. Convergence: Convex Case

In the presence of convexity, the proximal distance algorithm reduces to a proximal gradient algorithm. This follows from the representation

$$y=\overset{m}{\underset{i=1}{\sum }}\alpha_{i}P_{C_{i}}\left(x\right)=x-\overset{m}{\underset{i=1}{\sum }}\alpha_{i}\left[x-P_{C_{i}}\left(x\right)\right]=x-\nabla q\left(x\right)$$

involving the penalty *q*(***x***). Thus, the proximal distance algorithm can be expressed as

$$x_{k+1}={\text{prox}}_{\rho^{-1}f}\left[x_{k}-\nabla q\left(x_{k}\right)\right].$$

In this regard, there is the implicit assumption that *q*(***x***) is 1-smooth. This is indeed the case. According to the Moreau decomposition (Bauschke and Combettes, 2011), for a single closed convex set *C*

$$\nabla q\left(x\right)=x-P_{C}\left(x\right)={\text{prox}}_{\delta_{C}^{⋆}}\left(x\right),$$

where $\delta_{C}^{⋆}(x)$ is the Fenchel conjugate of the indicator function

$$\delta_{C}\left(x\right)=\{\begin{array}{ll} 0 & x\in C\\ \infty & x\notin C. \end{array}$$

Because proximal operators of closed convex functions are nonexpansive (Bauschke and Combettes, 2011), the result follows for a single set. For the general penalty *q*(***x***) with *m* sets, the Lipschitz constants are scaled by the convex coefficients *α*_*i*_ and added to produce an overall Lipschitz constant of 1.

It is enlightening to view the proximal distance algorithm through the lens of concaveconvex programming. Recall that the function

(8) $$s\left(x\right)=\underset{y\in C}{\text{sup}}\left[y^{t}x-\frac{1}{2}{\left\|y\right\|}^{2}\right]=\frac{1}{2}{\left\|x\right\|}^{2}-\frac{1}{2}\text{dist}{\left(x,C\right)}^{2}$$

is closed and convex for any nonempty closed set *C*. Danskin’s theorem (Lange, 2016) justifies the directional derivative expression

$$d_{v}s\left(x\right)=\underset{y\in P_{C}\left(x\right)}{\text{sup}}y^{t}v=\underset{y\in \text{conv}P_{C}\left(x\right)}{\text{sup}}y^{t}v.$$

This equality allows us to identify the subdifferential *∂s*(***x***) as the convex hull conv*P*_*C*_(*x*). For any ***y*** ∈ *∂s*(***x***_*k*_), the supporting hyperplane inequality entails

$$\frac{1}{2}\text{dist}{\left(x,C\right)}^{2}=\frac{1}{2}{\left\|x\right\|}^{2}-s(x)\;\leq \frac{1}{2}{\left\|x\right\|}^{2}-s\left(x_{k}\right)-y^{t}\left(x-x_{k}\right)\;=\frac{1}{2}{\left\|x-y\right\|}^{2}+d,$$

where *d* is a constant not depending on ***x***. The same majorization can be generated by rearranging the majorization

$$\frac{1}{2}\text{dist}{\left(x,C\right)}^{2}\leq \frac{1}{2}\underset{i}{\sum }\beta_{i}{\left\|x-p_{i}\right\|}^{2}$$

when ***y*** is the convex combination $\underset{i}{\sum }\beta_{i}p_{i}$ of vectors *p*_*i*_ from *P*_*C*_(***x***_*k*_). These facts demonstrate that the proximal distance algorithm minimizing

$$f\left(x\right)+\frac{\rho }{2}\text{dist}{\left(x,C\right)}^{2}=f\left(x\right)+\frac{\rho }{2}{\left\|x\right\|}^{2}-\rho s\left(x\right)$$

is a special case of concave-convex programming when *f*(***x***) is convex. It is worth emphasizing that $f(x)+\frac{\rho }{2}\|x{\|}^{2}$ is often strongly convex regardless of whether *f*(***x***) itself is convex. If we replace the penalty dist(***x****,C*)^2^ by the penalty dist(***Dx****,C*)^2^ for a matrix ***D***, then the function *s*(***Dx***) is still closed and convex, and minimization of $f(x)+\frac{\rho }{2}\text{dist}{\left(Dx,C\right)}^{2}$ can also be viewed as an exercise in concave-convex programming.

In the presence of convexity, the proximal distance algorithm is guaranteed to converge. Our exposition relies on well-known operator results (Bauschke and Combettes, 2011). Proximal operators in general and projection operators in particular are nonexpansive and averaged. By definition an averaged operator

M(x)=αx+(1−α)N(x)

is a convex combination of a nonexpansive operator *N*(***x***) and the identity operator ***I***. The averaged operators on ${\mathbb{R}}^{p}$ with *α* ∈ (0,1) form a convex set closed under functional composition. Furthermore, *M*(***x***) and the base operator *N*(***x***) share their fixed points. The celebrated theorem of Krasnosel’skii (1955) and Mann (1953) says that if an averaged operator *M*(***x***) = *α****x*** + (1 − *α*)*N*(***x***) possesses one or more fixed points, then the iteration scheme ***x***_*k*+1_ = *M*(***x***_*k*_) converges to a fixed point.

These results immediately apply to minimization of the penalized loss

(9) $$h_{\rho }\left(x\right)=f\left(x\right)+\frac{\rho }{2}\overset{m}{\underset{i=1}{\sum }}\alpha_{i}\text{dist}{\left(x,C_{i}\right)}^{2}.$$

Given the choice $y_{k}=\sum_{i=1}^{m}\alpha_{i}P_{C_{i}}\left(x_{k}\right)$, the algorithm map ***x***_*k*+1_ = prox_*ρ*_−_1*f*_(***y***_*k*_) is an averaged operator, being the composition of two averaged operators. Hence, the Krasnosel’skiiMann theorem guarantees convergence to a fixed point if one or more exist. Now *z* is a fixed point if and only if

$$h_{\rho }\left(z\right)\leq f\left(x\right)+\frac{\rho }{2}\overset{m}{\underset{i=1}{\sum }}\alpha_{i}{\left\|x-P_{C_{i}}\left(z\right)\right\|}^{2}$$

for all ***x***. In the presence of convexity, this is equivalent to the directional derivative inequality

$$0\leq d_{v}f\left(z\right)+\rho \overset{m}{\underset{i=1}{\sum }}\alpha_{i}{\left[z-P_{C_{i}}\left(z\right)\right]}^{t}v=d_{v}h_{\rho }\left(z\right)$$

for all ***v***, which is in turn equivalent to *z* minimizing *h*_*ρ*_(***x***). Hence, if *h*_*ρ*_(***x***) attains its minimum value, then the proximal distance iterates converge to a minimum point.

Convergence of the overall proximal distance algorithm is tied to the convergence of the classical penalty method (Beltrami, 1970). In our setting, the loss is *f*(***x***), and the penalty is $q(x)=\frac{1}{2}\sum_{i=1}^{m}\alpha_{i}\text{dist}{\left(x,C_{i}\right)}^{2}$. Assuming the objective *f*(***x***) + *ρq*(***x***) is coercive for all *ρ* ≥ 0, the theory mandates that the solution path ***x***_*ρ*_ is bounded and any limit point of the path attains the minimum value of *f*(***x***) subject to the constraints. Furthermore, if *f*(***x***) is coercive and possesses a unique minimum point in the constraint set *C*, then the path ***x***_*ρ*_ converges to that point.

Proximal distance algorithms often converge at a painfully slow rate. Following Mairal (2013), one can readily exhibit a precise bound.

**Proposition 1** *Suppose C is closed and convex and f*(***x***) *is convex. If the point z minimizes* $h_{\rho }(x)=f(x)+\frac{\rho }{2}\text{dist}{\left(x,C\right)}^{2}$, *then the proximal distance iterates satisfy*

$$0\leq h_{\rho }\left(x_{k+1}\right)-h_{\rho }\left(z\right)\leq \frac{\rho }{2(k+1)}{\left\|z-x_{0}\right\|}^{2}.$$

The *O*(*ρk*^−1^) convergence rate of the proximal distance algorithm suggests that one should slowly send *ρ* to ∞ and refuse to wait until convergence occurs for any given *ρ*. It also suggests that Nesterov acceleration may vastly improve the chances for convergence. Nesterov acceleration for the general proximal gradient algorithm with loss *ℓ*(***x***) and penalty *p*(***x***) takes the form

$$z_{k}=x_{k}+\frac{k-1}{k+d-1}\left(x_{k}-x_{k-1}\right)$$

(10) $$x_{k+1}={\text{prox}}_{L^{-1}l}\left[z_{k}-L^{-1}\nabla p\left(z_{k}\right)\right],$$

where *L* is the Lipschitz constant for ∇*p*(*x*) and *d* is typically chosen to be 3. Nesterov acceleration achieves an *O*(*k*^−2^) convergence rate (Su et al., 2014), which is vastly superior to the *O*(*k*^−1^) rate achieved by proximal gradient descent. The Nesterov update possesses the further desirable property of preserving affine constraints. In other words, if ***Ax***_*k*−1_ = ***b*** and ***Ax***_*k*_ = ***b***, then ***Az***_*k*_ = ***b*** as well. In subsequent examples, we will accelerate our proximal distance algorithms by applying the algorithm map *M*(***x***) given by equation (2) to the shifted point *z*_*k*_ of equation (10), yielding the accelerated update ***x***_*k*+1_ = *M*(*z*_*k*_). Algorithm 1 provides a schematic of a proximal distance algorithm with Nesterov acceleration. The recent paper of Ghadimi and Lan (2015) extends Nestorov acceleration to nonconvex settings.

In ideal circumstances, one can prove linear convergence of function values in the framework of Karimi et al. (2016).

**Proposition 2** *Suppose C is closed and convex and f*(***x***) *is L-smooth and μ-strongly convex. Then* $h_{\rho }(x)=f(x)+\frac{\rho }{2}\text{dist}{\left(x,C\right)}^{2}$ *possesses a unique minimum point* ***y****, and the proximal* distance iterates **x**_k_ satisfy

$$h_{\rho }\left(x_{k}\right)-h_{\rho }\left(y\right)\leq {\left(1-\frac{\mu^{2}}{2{\left(L+\rho \right)}^{2}}\right)}^{k}\left[h_{\rho }\left(x_{0}\right)-h_{\rho }\left(y\right)\right].$$

We now turn to convergence of the penalty function iterates as the penalty constants *ρ*_*k*_ tends to ∞. To simplify notation, we restrict attention to a single closed constraint set *S*. Let us start with a proposition requiring no convexity assumptions.

**Proposition 3** *If f*(***x***) *is continuous and coercive and S is compact, then the proximal distance iterates* ***x***_*k*_ *are bounded and the distance to the constraint set satisfies*

$$\text{dist}{\left(x_{k},S\right)}^{2}\leq \frac{c}{\rho_{k}}$$

*for some constant c. If in addition f*(***x***) *is continuously differentiable, then*

$$\text{dist}{\left(x_{k},S\right)}^{2}\leq \frac{d}{\rho_{k}^{2}}$$

for some further constant d. Similar claims hold for the solutions **y**_k_ of the penalty problem ${\text{min}}_{x}f(x)+\frac{\rho_{k}}{2}\text{dist}{\left(x,S\right)}^{2}$ except that the assumption that S is compact can be dropped.

As a corollary, if the penalty sequence *ρ*_*k*_ tends to ∞, then all limit points of ***x***_*k*_ must obey the constraint. Proposition 3 puts us into position to prove the next important result.

**Proposition 4** *If f*(***x***) *is continuously differentiable and coercive and S is convex, then the penalty function iterates defined by* $y_{k}\in {\text{argmin}}_{x}\left[f(x)+\frac{\rho_{k}}{2}\text{dist}{\left(x,S\right)}^{2}\right]$ *satisfy*

$$0\leq f\left(y\right)-f\left(y_{k}\right)\leq \frac{d+2\sqrt{d}\|\nabla f\left(y\right)\|}{2\rho_{k}},$$

where **y** attains the constrained minimum and d is the constant identified in Proposition 3.

## 4. Convergence: General Case

Our strategy for addressing convergence in nonconvex problems fixes *ρ* and relies on Zangwill’s global convergence theorem (Luenberger and Ye, 1984). This result depends in turn on the notion of a closed multi-valued map ***N***(***x***). If ***x***_*k*_ converges to ***x***_∞_ and ***y***_*k*_ ∈ *N*(***x***_*k*_) converges to ***y***_∞_, then for ***N***(***x***) to be closed, we must have ***y***_∞_ ∈ *N*(***x***_∞_). The next proposition furnishes a prominent example.

**Proposition 5** *If S is a closed nonempty set in* ${\mathbb{R}}^{p}$, *then the projection operator P*_*S*_(***x***) *is closed. Furthermore, if the sequence* ***x***_*k*_ *is bounded, then the set* ∪_*k*_*P*_*S*_(***x***_*k*_) *is bounded as well*.


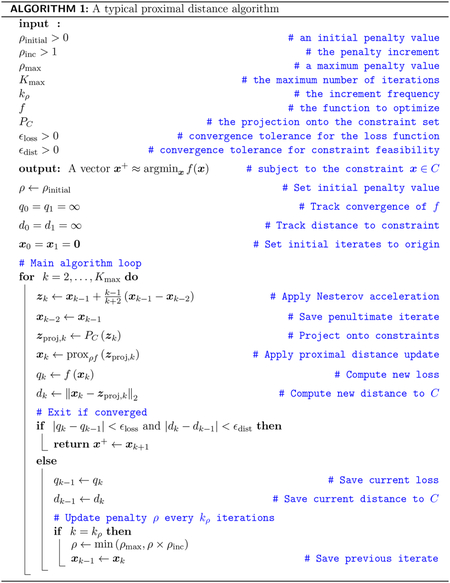


Zangwill’s global convergence theorem is phrased in terms of an algorithm map *M*(***x***) and a real-valued objective *h*(***x***). The theorem requires a critical set Γ outside which *M*(***x***) is closed. Furthermore, all iterates ***x***_*k*+1_ ∈ *M*(***x***_*k*_) must fall within a compact set. Finally, the descent condition *h*(***y***) ≤ *h*(***x***) should hold for all ***y*** ∈ *M*(***x***), with strict inequality when ***x*** ∉ Γ. If these conditions are valid, then every convergent subsequence of ***x***_*k*_ tends to a point in Γ. In the proximal distance context, we define the complement of Γ to consist of the points ***x*** with

$$f\left(y\right)+\frac{\rho }{2}\text{dist}{\left(y,S\right)}^{2}<f\left(x\right)+\frac{\rho }{2}\text{dist}{\left(x,S\right)}^{2}$$

for all ***y*** ∈ *M*(***x***). This definition plus the monotonic nature of the proximal distance algorithm

$$x_{k+1}\in M\left(x_{k}\right)=\underset{z_{k}\in P_{S}\left(x_{k}\right)}{\cup }{\text{argmin}}_{x}\left[f\left(x\right)+\frac{\rho }{2}{\left\|x-z_{k}\right\|}^{2}\right]$$

force the satisfaction of Zangwill’s final requirement. Note that if *f*(***x***) is differentiable, then a point ***x*** belongs to Γ whenever **0** ∈ ∇*f*(*x*) + *ρx* − *ρP*_*S*_(*x*).

In general, the algorithm map *M*(*x*) is multi-valued in two senses. First, for a given *z*_*k*_ ∈ *P*_*S*_(***x***_*k*_), the minimum may be achieved at multiple points. This contingency is ruled out if the proximal map of *f*(***x***) is unique. Second, because *S* may be nonconvex, the projection may be multi-valued. This sounds distressing, but the points ***x***_*k*_ where this occurs are exceptionally rare. Accordingly, it makes no practical difference that we restrict the anchor points *z*_*k*_ to lie in *P*_*S*_(***x***_*k*_) rather than in conv*P*_*S*_(***x***_*k*_).

**Proposition 6** *If S is a closed nonempty set in* ${\mathbb{R}}^{p}$, *then the projection operator P*_*S*_(***x***) *is single valued except on a set of Lebesgue measure* 0.

In view of the preceding results, one can easily verify the next proposition.

**Proposition 7** *The algorithm map M*(*x*) *is everywhere closed*.

To apply Zangwill’s global convergence theory, we must in addition prove that the iterates *x*_*k*+1_ = *M*(*x*_*k*_) remain within a compact set. This is true whenever the objective is coercive since the algorithm is a descent algorithm. As noted earlier, the coercivity of *f*(***x***) is a sufficient condition. One can readily concoct other sufficient conditions. For example, if *f*(***x***) is bounded below, say nonnegative, and *S* is compact, then the objective is also coercive. Indeed, if *S* is contained in the ball of radius *r* about the origin, then

$$\left\|x\right\|\leq \left\|x-P_{S}\left(x\right)\right\|+\left\|P_{S}\left(x\right)\right\|\leq \text{dist}\left(x,S\right)+r,$$

which proves that dist(***x****,S*) is coercive. The next proposition summarizes these findings.

**Proposition 8** *If S is closed and nonempty, the objective* $f(x)+\frac{1}{2}\text{dist}{\left(x,S\right)}^{2}$ *is coercive, and the proximal operator* prox_*ρ*_−_1f_(*x*) *is everywhere nonempty, then all limit points of the iterates* ***x***_k+1_ ∈ *M*(***x***_*k*_) *of the proximal distance algorithm occur in the critical set* Γ.

This result is slightly disappointing. A limit point ***x*** could potentially exist with improvement in the objective for some but not all ***y*** ∈ conv*P*_*S*_(***x***). This fault is mitigated by the fact that *P*_*S*_(***x***) is almost always single valued. In common with other algorithms in nonconvex optimization, we also cannot rule out convergence to a local minimum or a saddlepoint. One can improve on Proposition 8 by assuming that the surrogates *g*_*ρ*_(***x*** | ***x***_*k*_) are all *μ*-strongly convex. This is a small concession to make because *ρ* is typically large. If *f*(***x***) is convex, then *g*_*ρ*_(***x*** | ***x***_*k*_) is *ρ*-strongly convex by definition. It is also worth noting that any convex MM surrogate *g*(***x*** | ***x***_*k*_) can be made *μ*-strongly convex by adding the viscosity penalty $\frac{\mu }{2}\|x-x_{k}{\|}^{2}$ majorizing 0. The addition of a viscosity penalty seldom complicates finding the next iterate *x*_*n*+1_ and has little impact on the rate of convergence when *μ >* 0 is small.

**Proposition 9** *Under the μ-strongly convexity assumption on the surrogates g*_*ρ*_(***x*** | ***x***_*k*_)*, the proximal distance iterates satisfy* lim_k→∞_‖*x*_k+1_ −*x*_*k*_‖ = 0. *As a consequence, the set of limit points is connected as well as closed. Furthermore, if each limit point is isolated, then the iterates converge to a critical point*.

Further progress requires even more structure. Fortunately, what we now pursue applies to generic MM algorithms. We start with the concept of a Fréchet subdifferential (Kruger, 2003). If *h*(***x***) is a function mapping ${\mathbb{R}}^{p}$ into ℝ∪{+∞}, then its Fréchet subdifferential at ***x*** ∈ dom *f* is the set

$$\partial^{F}h\left(x\right)=\left\{v:\underset{y\to x}{\text{liminf}}\frac{h\left(y\right)-h\left(x\right)-v^{t}\left(y-x\right)}{\left\|y-x\right\|}\geq 0\right\}.$$

The set *∂*^*F*^*h*(***x***) is closed, convex, and possibly empty. If *h*(***x***) is convex, then *∂*^*F*^*h*(***x***) reduces to its convex subdifferential. If *h*(***x***) is differentiable, then *∂*^*F*^*h*(***x***) reduces to its ordinary differential. At a local minimum ***x***, Fermat’s rule **0** ∈ *∂*^*F*^*h*(*x*) holds.

**Proposition 10** *In an MM algorithm, suppose that h*(***x***) *is coercive, that the surrogates g*(***x*** | ***x***_*k*_) *are differentiable, and that the algorithm map M*(***x***) *is closed. Then every limit point z of the MM sequence* ***x***_*k*_ *is critical in the sense that* **0** ∈ *∂*^*F*^ (−*h*)(*z*).

We will also need to invoke Łojasiewicz’s inequality. This deep result depends on some rather arcane algebraic geometry (Bierstone and Milman, 1988; Bochnak et al., 2013). It applies to semialgebraic functions and their more inclusive cousins semianalytic functions and subanalytic functions. For simplicity we focus on semialgebraic functions. The class of semialgebraic subsets of ${\mathbb{R}}^{p}$ is the smallest class that:

- contains all sets of the form {***x*** : *q*(***x***) > 0} for a polynomial *q*(***x***) in *p* variables,
- is closed under the formation of finite unions, finite intersections, and set complementation.

A function *a* : ${\mathbb{R}}^{p}\mapsto {\mathbb{R}}^{r}$ is said to be semialgebraic if its graph is a semialgebraic set of ${\mathbb{R}}^{p+r}$. The class of real-valued semialgebraic functions contains all polynomials *p*(***x***) and all 0/1 indicators of algebraic sets. It is closed under the formation of sums, products, absolute values, reciprocals when *a*(***x***) 6≠ 0, *n*th roots when *a*(*x*) ≥ 0, and maxima max{*a*(***x***)*, b*(***x***)} and minima min{*a*(***x***)*, b*(***x***)}. For our purposes, it is important to note that dist(***x****,S*) is a semialgebraic function whenever *S* is a semialgebraic set.

Łojasiewicz’s inequality in its modern form (Bolte et al., 2007) requires a function *h*(***x***) to be closed (lower semicontinuous) and subanalytic with a closed domain. If *z* is a critical point of *h*(***x***), then

$${\left|h\left(x\right)-h\left(z\right)\right|}^{\theta }\leq c\left\|v\right\|$$

for all *x* ∈ *B*_*r*_(*z*)∩dom*∂*^*F*^*h* satisfying *h*(***x***) > *h*(***z***) and all ***v*** in *∂*^*F*^*h*(***x***). Here the exponent *θ* ∈ [0,1), the radius *r*, and the constant *c* depend on *z*. This inequality is valid for semialgebraic functions since they are automatically subanalytic. We will apply Łojasiewicz’s inequality to the limit points of an MM algorithm. The next proposition is an elaboration and expansion of known results (Attouch et al., 2010; Bolte et al., 2007; Cui et al., 2018; Kang et al., 2015; Le Thi et al., 2009).

**Proposition 11** *In an MM algorithm suppose the objective h*(***x***) *is coercive, continuous, and subanalytic and all surrogates g*(***x*** | ***x***_*k*_) *are continuous, μ-strongly convex, and satisfy the L-smoothness condition*

$$\left\|\nabla g\left(a|x_{k}\right)-\nabla g\left(b|x_{k}\right)\right\|\leq L\left\|a-b\right\|$$

*on the compact set* {*x* : *h*(***x***) ≤ *h*(***x***_0_)}. *Then the MM iterates* ***x***_k+1_ = argmin_***x***_*g*(***x*** | ***x***_*k*_) *converge to a critical point*.

The last proposition applies to proximal distance algorithms. The loss *f*(***x***) must be subanalytic and differentiable with a locally Lipschitz gradient. Furthermore, all surrogates $g\left(x|x_{k}\right)=f(x)+\frac{\rho }{2}\|x-y_{k}{\|}^{2}$ should be coercive and *μ*-strongly convex. Finally, the constraints sets *S*_*i*_ should be subanalytic. Semialgebraic sets and functions will do. Under these conditions and regardless of how the projected points *P*_*Si*_(***x***) are chosen, the MM iterates are guaranteed to converge to a critical point.

## 5. Examples

The following examples highlight the versatility of proximal distance algorithms in a variety of convex and nonconvex settings. Programming details matter in solving these problems. Individual programs are not necessarily long, but care must be exercised in projecting onto constraints, choosing tuning schedules, folding constraints into the domain of the loss, implementing acceleration, and declaring convergence. All of our examples are coded in the Julia programming language. Whenever possible, competing software was run in the Julia environment via the Julia module MathProgBase (Dunning et al., 2017; Lubin and Dunning, 2015). The sparse PCA problem relies on the software of Witten et al. (Witten et al., 2009), which is coded in R. Convergence is tested at iteration *k* by the two criteria

$$\left|f\left(x_{k}\right)-f\left(x_{k-1}\right)\right|\leq \epsilon_{1}\left[\left|f\left(x_{k-1}\right)\right|+1\right]\text{ and dist}\left(x_{k},C\right)\leq \epsilon_{2},$$

where ϵ_1_=10^−6^ and ϵ_2_=10^−4^ are typical values. The number of iterations until convergence is about 1000 in most examples. This handicap is offset by the simplicity of each stereotyped update. Our code is available as supplementary material to this paper. Readers are encouraged to try the code and adapt it to their own examples.

### 5.1 Linear Programming

Two different tactics suggest themselves for constructing a proximal distance algorithm. The first tactic rolls the standard affine constraints ***Ax*** = ***b*** into the domain of the loss function *v*^*t*^*x*. The standard nonnegativity requirement ***x*** ≥ **0** is achieved by penalization. Let *x*_*k*_ be the current iterate and ***y***_*k*_ = (***x***_*k*_)_+_ be its projection onto ${\mathbb{R}}_{+}^{n}$. Derivation of the proximal distance algorithm relies on the Lagrangian

$$v^{t}x+\frac{\rho }{2}{\left\|x-y_{k}\right\|}^{2}+\lambda^{t}\left(Ax-b\right).$$

One can multiply the corresponding stationarity equation

$$0=v+\rho \left(x-y_{k}\right)+A^{t}\lambda$$

by ***A*** and solve for the Lagrange multiplier ***λ*** in the form

(11) $$\lambda ={\left(AA^{t}\right)}^{-1}\left(\rho Ay_{k}-\rho b-Av\right)$$

assuming ***A*** has full row rank. Inserting this value into the stationarity equation gives the MM update

(12) $$x_{k+1}=y_{k}-\frac{1}{\rho }v-A^{-}\left(Ay_{k}-b-\frac{1}{\rho }Av\right),$$

where ***A***^−^ = ***A***^*t*^(***AA***^*t*^)^−1^ is the pseudo-inverse of ***A***.

The second tactic folds the nonnegativity constraints into the domain of the loss. Let ***p***_*k*_ denote the projection of ***x***_*k*_ onto the affine constraint set ***Ax*** = ***b***. Fortunately, the surrogate function $v^{t}x+\frac{\rho }{2}\|x-p_{k}{\|}^{2}$ splits the parameters. Minimizing one component at a time gives the update *x*_*k*+1_ with components

(13) $$x_{k+1,j}=\text{max}\left\{p_{kj}-\frac{v_{j}}{\rho },0\right\}.$$

The projection ***p***_*k*_ can be computed via

(14) $$p_{k}=x_{k}-A^{-}\left(Ax_{k}-b\right),$$

where ***A***^−^ is again the pseudo-inverse of ***A***.

Table 1 compares the accelerated versions of these two proximal distance algorithms to two efficient solvers. The first is the open-source Splitting Cone Solver (SCS) (O’Donoghue et al., 2016), which relies on a fast implementation of ADMM. The second is the commercial Gurobi solver, which ships with implementations of both the simplex method and a barrier (interior point) method; in this example, we use its barrier algorithm. The first seven rows of the table summarize linear programs with dense data ***A***, ***b***, and *v*. The bottom six rows rely on random sparse matrices ***A*** with sparsity level 0.01. For dense problems, the proximal distance algorithms start the penalty constant *ρ* at 1 and double it every 100 iterations. Because we precompute and cache the pseudoinverse ***A***^−^ of ***A***, the updates (12) and (13) reduce to vector additions and matrix-vector multiplications.

For sparse problems the proximal distance algorithms update *ρ* by a factor of 1.5 every 50 iterations. To avoid computing large pseudoinverses, we appeal to the LSQR variant of the conjugate gradient method (Paige and Saunders, 1982b,a) to solve the linear systems (11) and (14). The optima of all four methods agree to about 4 digits of accuracy. It is hard to declare an absolute winner in these comparisons. Gurobi and SCS clearly perform better on low-dimensional problems, but the proximal distance algorithms are competitive as dimensions increase. PD1, the proximal distance algorithm over an affine domain, tends to be more accurate than PD2. If high accuracy is not a concern, then the proximal distance algorithms are easily accelerated with a more aggressive update schedule for *ρ*.

### 5.2. Constrained Least Squares

Constrained least squares programming subsumes constrained quadratic programming. A typical quadratic program involves minimizing the quadratic $\frac{1}{2}x^{t}Qx-p^{t}x$ subject to *x* ∈ *C* for a positive definite matrix *Q*. Quadratic programming can be reformulated as least squares by taking the Cholesky decomposition *Q* = *LL*^*t*^ of *Q* and noting that

$$\frac{1}{2}x^{t}Qx-p^{t}x=\frac{1}{2}{\left\|L^{-1}p-L^{t}x\right\|}^{2}-\frac{1}{2}{\left\|L^{-1}p\right\|}^{2}.$$

The constraint *x* ∈ *C* applies in both settings. It is particularly advantageous to reframe a quadratic program as a least squares problem when *Q* is already presented in factored form or when it is nearly singular (Bemporad, 2018). To simplify subsequent notation, we replace ***L***^*t*^ by the rectangular matrix ***A*** and ***L***^−1^***p*** by ***y***. The key to solving constrained least squares is to express the proximal distance surrogate as

$$\frac{1}{2}{\left\|y-Ax\right\|}^{2}+\frac{\rho }{2}{\left\|x-P_{C}\left(x_{k}\right)\right\|}^{2}=\frac{1}{2}{\left\|\left(\begin{array}{c} y\\ \sqrt{\rho }P_{C}\left(x_{k}\right) \end{array}\right)-\left(\begin{array}{c} A\\ \sqrt{\rho }I \end{array}\right)x\right\|}^{2}$$

as in equation (6). As noted earlier, in sparse problems the update ***x***_*k*+1_ can be found by a fast stable conjugate gradient solver.

Table 2 compares the performance of the proximal distance algorithm for least squares estimation with probability-simplex constraints to the open source nonlinear interior point solver Ipopt (Wächter and Biegler, 2005, 2006) and the interior point method of Gurobi. Simplex constrained problems arise in hyperspectral imaging (Heylen et al., 2011; Keshava, 2003), portfolio optimization (Markowitz, 1952), and density estimation (Bunea et al., 2010). Test problems were generated by filling an *n*×*p* matrix ***A*** and an *n*-vector ***y*** with standard normal deviates. For sparse problems we set the sparsity level of ***A*** to be 10*/p*. Our setup ensures that ***A*** has full rank and that the quadratic program has a solution. For the proximal distance algorithm, we start *ρ* at 1 and multiply it by 1.5 every 200 iterations. Table 2 suggests that the proximal distance algorithm and the interior point solvers perform equally well on small dense problems. However, in high-dimensional and low-accuracy environments, the proximal distance algorithm provides much better scalability.

### 5.3. Closest Kinship Matrix

In genetics studies, kinship is measured by the fraction of genes two individuals share identical by descent. For a given pedigree, the kinship coefficients for all pairs of individuals appear as entries in a symmetric kinship matrix ***Y***. This matrix possesses three crucial properties: a) it is positive semidefinite, b) its entries are nonnegative, and c) its diagonal entries are $\frac{1}{2}$ unless some pedigree members are inbred. Inbreeding is the exception rather than the rule. Kinship matrices can be estimated empirically from single nucleotide polymorphism (SNP) data, but there is no guarantee that the three highlighted properties are satisfied. Hence, it helpful to project ***Y*** to the nearest qualifying matrix.

This projection problem is best solved by folding the positive semidefinite constraint into the domain of the Frobenius loss function $\frac{1}{2}\|X-Y{\|}_{F}^{2}$. As we shall see, the alternative of imposing two penalties rather than one is slower and less accurate. Projection onto the constraints implied by conditions b) and c) is trivial. All diagonal entries *x*_*ii*_ of ***X*** are reset to $\frac{1}{2}$, and all off-diagonal entries *x*_*ij*_ are reset to max{*x*_*ij*_,0}. If *P*(***X***_*k*_) denotes the current projection, then the proximal distance algorithm minimizes the surrogate

$$g\left(X|X_{k}\right)=\frac{1}{2}{\left\|X-Y\right\|}_{F}^{2}+\frac{\rho }{2}{\left\|X-P\left(X_{k}\right)\right\|}_{F}^{2}\;=\frac{1+\rho }{2}{\left\|X-\frac{1}{1+\rho }Y-\frac{\rho }{1+\rho }P\left(X_{k}\right)\right\|}_{F}^{2}+c_{k},$$

where *c*_*k*_ is an irrelevant constant. The minimum is found by extracting the spectral decomposition ***UDU***^*t*^ of $\frac{1}{1+\rho }Y+\frac{\rho }{1+\rho }P\left(X_{k}\right)$ and truncating the negative eigenvalues. This gives the update ***X***_*k*+1_ = ***UD***_+_***U***^*t*^ in obvious notation. This proximal distance algorithm and its Nesterov acceleration are simple to implement in a numerically oriented language such as Julia. The most onerous part of the calculation is clearly the repeated eigen-decompositions.

Table 3 compares three versions of the proximal distance algorithm to Dykstra’s algorithm (Boyle and Dykstra, 1986). Higham proposed Dykstra’s algorithm for the related problem of finding the closest correlation matrix Higham (2002). In Table 3 algorithm PD1 is the unadorned proximal distance algorithm, PD2 is the accelerated proximal distance, and PD3 is the accelerated proximal distance algorithm with the positive semidefinite constraints folded into the domain of the loss. On this demanding problem, these algorithms are comparable to Dykstra’s algorithm in speed but slightly less accurate. Acceleration of the proximal distance algorithm is effective in reducing both execution time and error. Folding the positive semidefinite constraint into the domain of the loss function leads to further improvements. The data matrices ***M*** in these trials were populated by standard normal deviates and then symmetrized by averaging opposing off-diagonal entries. In algorithm PD1 we set *ρ*_*k*_ = max{1.2^*k*^,2^22^}. In the accelerated versions PD2 and PD3 we started *ρ* at 1 and multiplied it by 5 every 100 iterations. At the expense of longer compute times, better accuracy can be achieved by all three proximal distance algorithms with a less aggressive update schedule.

### 5.4. Projection onto a Second-Order Cone Constraint

Second-order cone programming is one of the unifying themes of convex analysis (Alizadeh and Goldfarb, 2003; Lobo et al., 1998). It revolves around conic constraints of the form {***u*** : ‖*Au* + *b*‖ ≤ *c*^*t*^***u*** + *d*}. Projection of a vector ***x*** onto such a constraint is facilitated by parameter splitting. In this setting parameter splitting introduces a vector ***w***, a scalar *r*, and the two affine constraints ***w*** = ***Au*** + ***b*** and *r* = ***c***^*t*^***u*** + *d*. The conic constraint then reduces to the Lorentz cone constraint ‖*w*‖ ≤ *r*, for which projection is straightforward (Boyd and Vandenberghe, 2009). If we concatenate the parameters into the single vector

$$y=\left(\begin{array}{l} u\\ w\\ r \end{array}\right)$$

and define *L* = {***y*** : ‖*w*‖ ≤ *r*} and *M* = {***y*** : ***w*** = ***Au*** + ***b*** and *r* = ***c***^*t*^***u*** + *d*}, then we can rephrase the problem as minimizing $\frac{1}{2}\|x-u{\|}^{2}$ subject to ***y*** ∈ *L* ∩ *M*. This is a fairly typical set projection problem except that the ***w*** and *r* components of *y* are missing in the loss function.

Taking a cue from Example 5.1, we incorporate the affine constraints in the domain of the objective function. If we represent projection onto *L* by

$$P\left(\begin{array}{c} w_{k}\\ r_{k} \end{array}\right)=\left(\begin{array}{c} {\tilde{w}}_{k}\\ {\tilde{r}}_{k} \end{array}\right),$$

then the Lagrangian generated by the proximal distance algorithm amounts to

$$L=\frac{1}{2}\|x-u{\|}^{2}+\frac{\rho }{2}\|\left(\begin{array}{c} w-{\tilde{w}}_{k}\\ r-{\tilde{r}}_{k} \end{array}\right){\|}^{2}+\lambda^{t}(Au+b-w)+\theta \left(c^{t}u+d-r\right).$$

This gives rise to a system of three stationarity equations

(15) $$0=u-x+A^{t}\lambda +\theta c$$

(16) $$0=\rho \left(w-{\tilde{w}}_{k}\right)-\lambda$$

(17) $$0=\rho \left(r-{\tilde{r}}_{k}\right)-\theta .$$

Solving for the multipliers ***λ*** and *θ* in equations (16) and (17) and substituting their values in equation (15) yield

$$0=u-x+\rho A^{t}\left(w-{\tilde{w}}_{k}\right)+\rho \left(r-{\tilde{r}}_{k}\right)c\;=u-x+\rho A^{t}\left(Au+b-{\tilde{w}}_{k}\right)+\rho \left(c^{t}u+d-{\tilde{r}}_{k}\right)c.$$

This leads to the MM update

(18) $$u_{k+1}={\left(\rho^{-1}I+A^{t}A+cc^{t}\right)}^{-1}\left[\rho^{-1}x+A^{t}\left({\tilde{w}}_{k}-b\right)+\left({\tilde{r}}_{k}-d\right)c\right].$$

The updates ***w***_*k*+1_ = ***Au***_*k*+1_ + ***b*** and *r*_*k*+1_ = ***c***^*t*^***u***_*k*+1_ + *d* follow from the constraints.

Table 4 compares the proximal distance algorithm to SCS and Gurobi. Echoing previous examples, we tailor the update schedule for *ρ* differently for dense and sparse problems. Dense problems converge quickly and accurately when we set *ρ*_0_ = 1 and double *ρ* every 100 iterations. Sparse problems require a greater range and faster updates of *ρ*, so we set *ρ*_0_ = 0.01 and then multiply *ρ* by 2.5 every 10 iterations. For dense problems, it is clearly advantageous to cache the spectral decomposition of ***A***^*t*^***A*** + ***cc***^*t*^ as suggested in Example 5.2. In this regime, the proximal distance algorithm is as accurate as Gurobi and nearly as fast. SCS is comparable to Gurobi in speed but notably less accurate.

With a large sparse constraint matrix ***A***, extraction of its spectral decomposition becomes prohibitive. If we let ***E*** = (*ρ*^−1*/*2^***I A***^*t*^ *c*), then we must solve a linear system of equations defined by the Gramian matrix ***G*** = ***EE***^*t*^. There are three reasonable options for solving this system. The first relies on computing and caching a sparse Cholesky decomposition of ***G***. The second computes the QR decomposition of the sparse matrix ***E***. The R part of the QR decomposition coincides with the Cholesky factor. Unfortunately, every time *ρ* changes, the Cholesky or QR decomposition must be redone. The third option is the conjugate gradient algorithm. In our experience the QR decomposition offers superior stability and accuracy. When ***E*** is very sparse, the QR decomposition is often much faster than the Cholesky decomposition because it avoids forming the dense matrix ***A***^*t*^***A***. Even when only 5% of the entries of ***A*** are nonzero, 90% of the entries of ***A***^*t*^***A*** can be nonzero. If exquisite accuracy is not a concern, then the conjugate gradient method provides the fastest update. Table 4 reflects this choice.

### 5.5. Copositive Matrices

A symmetric matrix ***M*** is copositive if its associated quadratic form ***x***^*t*^***Mx*** is nonnegative for all *x* ≥ **0**. Copositive matrices find applications in numerous branches of the mathematical sciences (Berman and Plemmons, 1994). All positive semidefinite matrices and all matrices with nonnegative entries are copositive. The variational index

$$\mu \left(M\right)=\underset{\left\|x\right\|=1,x\geq 0}{\text{min}}x^{t}Mx$$

is one key to understanding copositive matrices (Hiriart-Urruty and Seeger, 2010). The constraint set *S* is the intersection of the unit sphere and the nonnegative cone ${\mathbb{R}}_{+}^{n}$. Projection of an external point ***y*** onto *S* splits into three cases. When all components of ***y*** are negative, then *P*_*S*_(***y***) = *e*_*i*_, where *y*_*i*_ is the least negative component of ***y***, and ***e***_*i*_ is the standard unit vector along coordinate direction *i*. The origin **0** is equidistant from all points of *S*. If any component of ***y*** is positive, then the projection is constructed by setting the negative components of ***y*** equal to 0, and standardizing the truncated version of ***y*** to have Euclidean norm 1.

As a test case for the proximal distance algorithm, consider the Horn matrix (Hall and Newman, 1963)

$$M=\left[\begin{array}{rrrrr} 1 & -1 & 1 & 1 & -1\\ -1 & 1 & -1 & 1 & 1\\ 1 & -1 & 1 & -1 & 1\\ 1 & 1 & -1 & 1 & -1\\ -1 & 1 & 1 & -1 & 1 \end{array}\right].$$

The value *μ*(*M*) = 0 is attained for the vectors $\frac{1}{\sqrt{2}}{\left(1,1,0,0,0\right)}^{t}$, $\frac{1}{\sqrt{6}}{\left(1,2,1,0,0\right)}^{t}$, and equivalent vectors with their entries permuted. Matrices in higher dimensions with the same Horn pattern of 1’s and −1’s are copositive as well (Johnson and Reams, 2008). A Horn matrix of odd dimension cannot be written as a positive semidefinite matrix, a nonnegative matrix, or a sum of two such matrices.

The proximal distance algorithm minimizes the criterion

$$g\left(x|x_{k}\right)=\frac{1}{2}x^{t}Mx+\frac{\rho }{2}{\left\|x-P_{S}\left(x_{k}\right)\right\|}^{2}$$

and generates the updates

$$x_{k+1}={\left(M+\rho I\right)}^{-1}\rho P_{S}\left(x_{k}\right).$$

It takes a gentle tuning schedule to get decent results. The choice *ρ*_*k*_ = 1.2^*k*^ converges in 600 to 700 iterations from random starting points and reliably yields objective values below 10^−5^ for Horn matrices. The computational burden per iteration is significantly eased by exploiting the cached spectral decomposition of ***M***. Table 5 compares the performance of the proximal distance algorithm to the Mosek solver on a range of Horn matrices. Mosek uses semidefinite programming to decide whether ***M*** can be decomposed into a sum of a positive semidefinite matrix and a nonnegative matrix. If not, Mosek declares the problem infeasible. Nesterov acceleration improves the final loss for the proximal distance algorithm, but it does not decrease overall computing time.

Testing for copositivity is challenging because neither the loss function nor the constraint set is convex. The proximal distance algorithm offers a fast screening device for checking whether a matrix is copositive. On random 1000×1000 symmetric matrices ***M***, the method invariably returns a negative index in less than two seconds of computing time. Because the vast majority of symmetric matrices are not copositive, accurate estimation of the minimum is not required. Table 6 summarizes a few random trials with lower-dimensional symmetric matrices. In higher dimensions, Mosek becomes non-competitive, and Nesterov acceleration is of dubious value.

### 5.6. Linear Complementarity Problem

The linear complementarity problem (Murty and Yu, 1988) consists of finding vectors *x* and *y* with nonnegative components such that ***x***^*t*^***y*** = 0 and ***y*** = ***A****x* + ***b*** for a given square matrix *A* and vector *b*. The natural loss function is $\frac{1}{2}\|y-Ax-b{\|}^{2}$. To project a vector pair (***u***,***v***) onto the nonconvex constraint set, one considers each component pair (*u*_*i*_*, v*_*i*_) in turn. If *u*_*i*_ ≥ max{*v*_*i*_,0}, then the nearest pair (***x***,***y***) has components (*x*_*i*_*, y*_*i*_) = (*u*_*i*_,0). If *v*_*i*_ ≥ max{*u*_*i*_,0}, then the nearest pair has components (*x*_*i*_*, y*_*i*_) = (0*, v*_*i*_). Otherwise, (*x*_*i*_*, y*_*i*_) = (0,0). At each iteration the proximal distance algorithm minimizes the criterion

$$\frac{1}{2}{\left\|y-Ax-b\right\|}^{2}+\frac{\rho }{2}{\left\|x-{\tilde{x}}_{k}\right\|}^{2}+\frac{\rho }{2}{\left\|y-{\tilde{y}}_{k}\right\|}^{2},$$

where (${\tilde{x}}_{k}$, ${\tilde{y}}_{k}$) is the projection of (***x***_*k*_, ***y***_*k*_) onto the constraint set. The stationarity equations become

$$0=-A^{t}\left(y-Ax-b\right)+\rho \left(x-{\tilde{x}}_{k}\right)$$

$$0=y-Ax-b+\rho \left(y-{\tilde{y}}_{k}\right).$$

Substituting the value of ***y*** from the second equation into the first equation leads to the updates

(19) $$x_{k+1}={\left[(1+\rho )I+A^{t}A\right]}^{-1}\left[A^{t}\left({\tilde{y}}_{k}-b\right)+(1+\rho ){\tilde{x}}_{k}\right]$$

$$y_{k+1}=\frac{1}{1+\rho }\left(Ax_{k+1}+b\right)+\frac{\rho }{1+\rho }{\tilde{y}}_{k}.$$

The linear system (19) can be solved in low to moderate dimensions by computing and caching the spectral decomposition of ***A***^*t*^***A*** and in high dimensions by the conjugate gradient method. Table 7 compares the performance of the proximal gradient algorithm to the Gurobi solver on some randomly generated problems.

### 5.7. Sparse Principal Components Analysis

Let ***X*** be an *n* × *p* data matrix gathered on *n* cases and *p* predictors. Assume the columns of ***X*** are centered to have mean 0. Principal component analysis (PCA) (Hotelling, 1933; Pearson, 1901) operates on the sample covariance matrix $S=\frac{1}{n}X^{t}X$. Here we formulate a proximal distance algorithm for sparse PCA (SPCA), which has attracted substantial interest in the machine learning community (Berthet and Rigollet, 2013b,a; D’Aspremont et al., 2007; Johnstone and Lu, 2009; Journée et al., 2010; Witten et al., 2009; Zou et al., 2006). According to a result of Ky Fan (Fan, 1949), the first *q* principal components (PCs) ***u***_1_,…,***u***_*q*_ can be extracted by maximizing the function tr(***U***^*t*^***SU***) subject to the matrix constraint ***U***^*t*^***U*** = ***I***_*q*_, where ***u***_*i*_ is the *i*th column of the *p*×*q* matrix ***U***. This constraint set is called a Stiefel manifold. One can impose sparsity by insisting that any given column ***u***_*i*_ have at most *r* nonzero entries. Alternatively, one can require the entire matrix *U* to have at most *r* nonzero entries. The latter choice permits sparsity to be distributed non-uniformly across columns.

Extraction of sparse PCs is difficult for three reasons. First, the Stiefel manifold ***M***_*q*_ and both sparsity sets are nonconvex. Second, the objective function is concave rather than convex. Third, there is no simple formula or algorithm for projecting onto the intersection of the two constraint sets. Fortunately, it is straightforward to project onto each separately. Let $P_{M_{q}}(U)$ denote the projection of ***U*** onto the Stiefel manifold. It is well known that $P_{M_{q}}(U)$ can be calculated by extracting a partial singular value decomposition ***U*** = ***V*Σ*W***^*t*^ of ***U*** and setting *P*_*Mq*_(***U***) = ***VW***^*t*^ (Golub and Van Loan, 2012). Here ***V*** and ***W*** are orthogonal matrices of dimension *p*×*q* and *q*×*q*, respectively, and **Σ** is a diagonal matrix of dimension *q* × *q*. Let $P_{S_{r}}(U)$ denote the projection of ***U*** onto the sparsity set

$$S_{r}=\left\{V:v_{ij}\neq 0\text{ for at most }r\text{ entries of each column }v_{i}\right\}.$$

Because $P_{S_{r}}(U)$ operates column by column, it suffices to project each column vector ***u***_*i*_ to sparsity. This entails nothing more than sorting the entries of ***u***_*i*_ by magnitude, saving the *r* largest, and sending the remaining *p*−*r* entries to 0. If the entire matrix ***U*** must have at most *r* nonzero entries, then ***U*** can be treated as a concatenated vector during projection.

The key to a good algorithm is to incorporate the Stiefel constraints into the domain of the objective function (Kiers, 1990; Kiers and ten Berge, 1992) and the sparsity constraints into the distance penalty. Thus, we propose decreasing the criterion

$$f\left(U\right)=-\frac{1}{2}\text{tr}\left(U^{t}SU\right)+\frac{\rho }{2}\text{dist}{\left(U,S_{r}\right)}^{2}.$$

at each iteration subject to the Stiefel constraints. The loss can be majorized via

$$-\frac{1}{2}\text{tr}\left(U^{t}SU\right)=-\frac{1}{2}\text{tr}\left[{\left(U-U_{k}\right)}^{t}S\left(U-U_{k}\right)\right]-\text{tr}\left(U^{t}SU_{k}\right)+\frac{1}{2}\text{tr}\left(U_{k}^{t}SU_{k}\right)\;\leq -\text{tr}\left(U^{t}SU_{k}\right)+\frac{1}{2}\text{tr}\left(U_{k}^{t}SU_{k}\right)$$

because ***S*** is positive semidefinite. The penalty is majorized by

$$\frac{\rho }{2}\text{dist}{\left(U,S_{r}\right)}^{2}\leq -\rho \text{tr}\left[U^{t}P_{S_{r}}\left(U_{k}\right)\right]+c_{k}$$

up to an irrelevant constant *c*_*k*_ since the squared Frobenius norm satisfies the relation $\|U^{t}U{\|}_{F}^{2}=q$ on the Stiefel manifold. It now follows that *f*(***U***) is majorized by

$$\frac{1}{2}{\left\|U-SU_{k}-\rho P_{S_{r}}\left(U_{k}\right)\right\|}_{F}^{2}$$

up to an irrelevant constant. Accordingly, the Stiefel projection

$$U_{k+1}=P_{M_{q}}\left[SU_{k}+\rho P_{S_{r}}\left(U_{k}\right)\right]$$

provides the next MM iterate.

**Figure 1:**
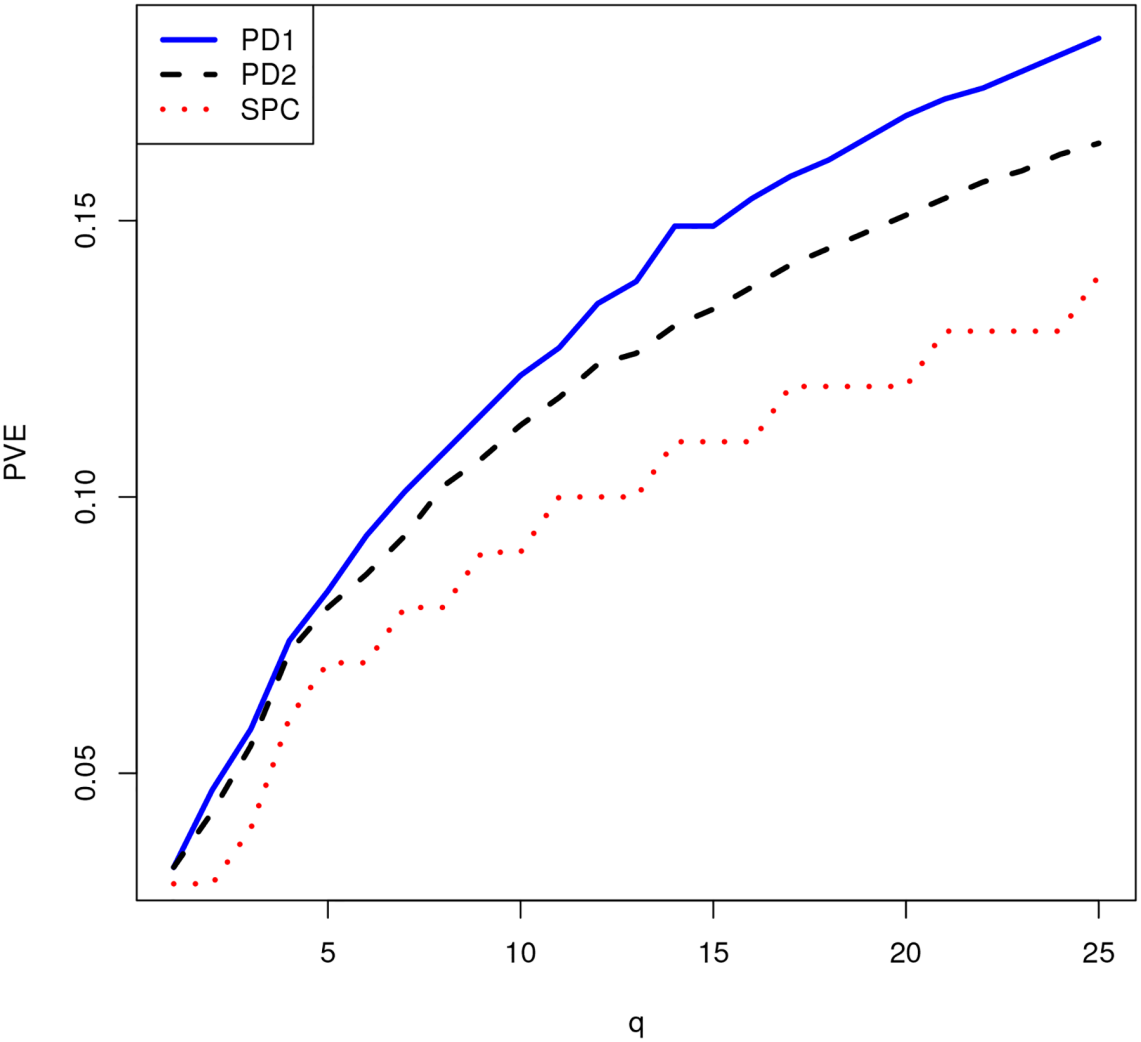
Proportion of variance explained by *q* PCs for each algorithm. Here PD1 is the accelerated proximal distance algorithm enforcing matrix sparsity, PD2 is the accelerated proximal distance algorithm enforcing column-wise sparsity, and SPC is the orthogonal sparse PCA method from PMA.

**Figure 2:**
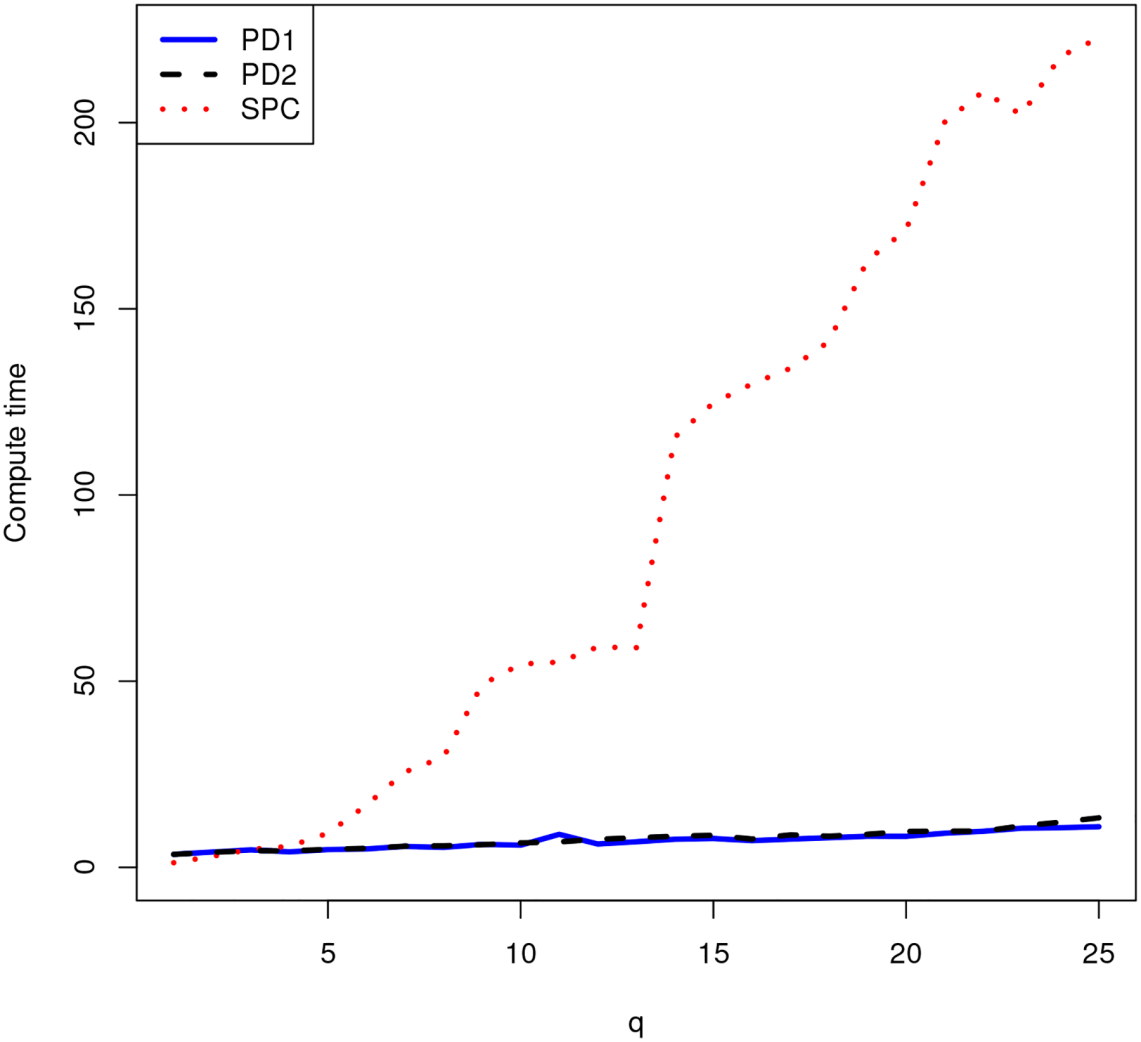
Computation times for *q* PCs for each algorithm. Here PD1 is the accelerated proximal distance algorithm enforcing matrix sparsity, PD2 is the accelerated proximal distance algorithm enforcing column-wise sparsity, and SPC is the orthogonal sparse PCA method from PMA.

Figures 1 and 2 compare the proximal distance algorithm to the SPC function from the R package PMA (Witten et al., 2009). The breast cancer data from PMA provide the data matrix ***X***. The data consist of *p* = 19,672 RNA measurements on *n* = 89 patients. The two figures show computation times and the proportion of variance explained (PVE) by the *p* × *q* loading matrix ***U***. For sparse PCA, PVE is defined as $\text{tr}\left(X_{q}^{t}X_{q}\right)/\text{tr}\left(X^{t}X\right)$, where ***X***_*q*_ = ***XU***(***U***^*t*^***U***)^−1^***U***^*t*^ (Shen and Huang, 2008). When the loading vectors of ***U*** are orthogonal, this criterion reduces to the familiar definition tr(***U***^*t*^***X***^*t*^***XU***)*/*tr(***X***^*t*^***X***) of PVE for ordinary PCA. The proximal distance algorithm enforces either matrix-wise or column-wise sparsity. In contrast, SPC enforces only column-wise sparsity via the constraint ‖*u*_*i*_‖_1_ ≤ *c* for each column ***u***_*i*_ of ***U***. We take *c* = 8. The number of nonzeroes per loading vector output by SPC dictates the sparsity level for the column-wise version of the proximal distance algorithm. Summing these counts across all columns dictates the sparsity level for the matrix version of the proximal distance algorithm.

Figures 1 and 2 demonstrate the superior PVE and computational speed of both proximal distance algorithms versus SPC. The type of projection does not appear to affect the computational performance of the proximal distance algorithm, as both versions scale equally well with *q*. However, the matrix projection, which permits the algorithm to more freely assign nonzeroes to the loadings, attains better PVE than the more restrictive column-wise projection. For both variants of the proximal distance algorithm, Nesterov acceleration improves both fitting accuracy and computational speed, especially as the number of PCs *q* increases.

## 6. Discussion

The proximal distance algorithm applies to a host of problems. In addition to the linear and quadratic programming examples considered here, our previous paper (Lange and Keys, 2014) derives and tests algorithms for binary piecewise-linear programming, *ℓ*_0_ regression, matrix completion (Cai et al., 2010; Candès and Tao, 2010; Chen et al., 2012; Mazumder et al., 2010), and sparse precision matrix estimation (Friedman et al., 2008). Other potential applications immediately come to mind. An integer linear program in standard form can be expressed as minimizing *c*^*t*^*x* subject to ***Ax*** + ***s*** = ***b***, ***s*** ≥ **0**, and $x\in {\mathbb{Z}}^{p}$. The latter two constraints can be combined in a single constraint for which projection is trivial. The affine constraints should be folded into the domain of the objective. Integer programming is NP hard, so that the proximal distance algorithm just sketched is merely heuristic. Integer linear programming includes traditional NP hard problems such as the traveling salesman problem, the vertex cover problem, set packing, and Boolean satisfiability. It will be interesting to see if the proximal distance principle is competitive in meeting these challenges. Our experience with the closest lattice point problem (Agrell et al., 2002) and the eight queens problem suggests that the proximal distance algorithm can be too greedy for hard combinatorial problems. The nonconvex problems solved in this paper are in some vague sense easy combinatorial problems.

The behavior of a proximal distance algorithm depends critically on a sensible tuning schedule for increasing *ρ*. Starting *ρ* too high puts too much stress on satisfying the constraints. Incrementing *ρ* too quickly causes the algorithm to veer off the solution path guaranteed by the penalty method. Given the chance of roundoff error even with double precision arithmetic, it is unwise to take *ρ* all the way to ∞. Trial and error can help in deciding whether a given class of problems will benefit from an aggressive update schedule and strict or loose convergence criteria. In problems with little curvature such as linear programming, more conservative updates are probably prudent. The linear programming, closest kinship matrix, and SPCA problems document the value of folding constraints into the domain of the loss. In the same spirit it is wise to minimize the number of constraints. A single penalty for projecting onto the intersection of two constraint sets is almost always preferable to the sum of two penalties for their separate projections. Exceptions to this rule obviously occur when projection onto the intersection is intractable. The integer linear programming problem mentioned previously illustrates these ideas.

Our earlier proximal distance algorithms ignored acceleration. In many cases the solutions produced had very low accuracy. The realization that convex proximal distance algorithms can be phrased as proximal gradient algorithms convinced us to try Nesterov acceleration. We now do this routinely on the subproblems with *ρ* fixed. This typically forces tighter path following and a reduction in overall computing times. Our examples generally bear out the contention that Nesterov acceleration is useful in nonconvex problems (Ghadimi and Lan, 2015). It is noteworthy that the value of acceleration often lies in improving the quality of a solution as much as it does in increasing the rate of convergence. Of course, acceleration cannot prevent convergence to an inferior local minimum.

On both convex and nonconvex problems, proximal distance algorithms enjoy global convergence guarantees. On nonconvex problems, one must confine attention to subanalytic sets and subanalytic functions. This minor restriction is not a handicap in practice. Determining local convergence rates is a more vexing issue. For convex problems, we review existing theory for a fixed penalty constant *ρ*. The classical results buttress an *O*(*ρk*^−1^) sublinear rate for general convex problems. Better results require restrictive smoothness assumptions on both the objective function and the constraint sets. For instance, when *f*(***x***) is *L*-smooth and strongly convex, linear convergence can be demonstrated. When *f*(***x***) equals a difference of convex functions, proximal distance algorithms reduce to concave-convex programming. Le Thi et al. (2009) attack convergence in this setting.

We hope readers will sense the potential of the proximal distance principle. This simple idea offers insight into many existing algorithms and a straightforward path in devising new ones. Effective proximal and projection operators usually spell the difference between success and failure. The number and variety of such operators is expanding quickly as the field of optimization relinquishes it fixation on convexity. The current paper research leaves many open questions about tuning schedules, rates of convergence, and acceleration in the face of nonconvexity. We welcome the contributions of other mathematical scientists in unraveling these mysteries and in inventing new proximal distance algorithms.

## Supplementary Material

code

## Figures and Tables

**Table 1:** CPU times and optima for linear programming. Here *m* is the number of constraints, *n* is the number of variables, PD1 is the proximal distance algorithm over an affine domain, PD2 is the proximal distance algorithm over a nonnegative domain, SCS is the Splitting Cone Solver, and Gurobi is the Gurobi solver. After *m* = 512 the constraint matrix ***A*** is initialized to be sparse with sparsity level *s* = 0.01.

Dimensions		Optima				CPU Times (secs)			
*m*	*n*	PD1	PD2	SCS	Gurobi	PD1	PD2	SCS	Gurobi
2	4	0.2629	0.2629	0.2629	0.2629	0.0142	0.0010	0.0034	0.0038
4	8	1.0455	1.0457	1.0456	1.0455	0.0212	0.0021	0.0009	0.0011
8	16	2.4513	2.4515	2.4514	2.4513	0.0361	0.0048	0.0018	0.0029
16	32	3.4226	3.4231	3.4225	3.4223	0.0847	0.0104	0.0090	0.0036
32	64	6.2398	6.2407	6.2397	6.2398	0.1428	0.0151	0.0140	0.0055
64	128	14.671	14.674	14.671	14.671	0.2117	0.0282	0.0587	0.0088
128	256	27.116	27.125	27.116	27.116	0.3993	0.0728	0.8436	0.0335
256	512	58.501	58.512	58.494	58.494	0.7426	0.1538	2.5409	0.1954
512	1024	135.35	135.37	135.34	135.34	1.6413	0.5799	5.0648	1.7179
1024	2048	254.50	254.55	254.47	254.48	2.9541	3.2127	3.9433	0.6787
2048	4096	533.29	533.35	533.23	533.23	7.3669	17.318	25.614	5.2475
4096	8192	991.78	991.88	991.67	991.67	30.799	95.974	98.347	46.957
8192	16384	2058.8	2059.1	2058.5	2058.5	316.44	623.42	454.23	400.59

**Table 2:** CPU times and optima for simplex-constrained least squares. Here $A\in {\mathbb{R}}^{n\times p}$, PD is the proximal distance algorithm, IPOPT is the Ipopt solver, and Gurobi is the Gurobi solver. After *n* = 1024, the predictor matrix *A* is sparse.

Dimensions		Optima			CPU Times		
*n*	*p*	PD	IPOPT	Gurobi	PD	IPOPT	Gurobi
16	8	4.1515	4.1515	4.1515	0.0038	0.0044	0.0010
32	16	10.8225	10.8225	10.8225	0.0036	0.0039	0.0010
64	32	29.6218	29.6218	29.6218	0.0079	0.0079	0.0019
128	64	43.2626	43.2626	43.2626	0.0101	0.0078	0.0033
256	128	111.7642	111.7642	111.7642	0.0872	0.0151	0.0136
512	256	231.6455	231.6454	231.6454	0.1119	0.0710	0.0619
1024	512	502.1276	502.1276	502.1276	0.2278	0.4013	0.2415
2048	1024	994.2447	994.2447	994.2447	1.2575	2.3346	1.1682
4096	2048	2056.8381	2056.8381	2056.8381	1.3253	15.2214	7.4971
8192	4096	4103.4611	4103.4611	4103.4611	3.0289	146.1604	49.7411
16384	8192	8295.2136	8295.2136	8295.2136	6.8739	732.1039	412.3612

**Table 3:** CPU times and optima for the closest kinship matrix problem. Here the kinship matrix is *n* × *n*, PD1 is the proximal distance algorithm, PD2 is the accelerated proximal distance, PD3 is the accelerated proximal distance algorithm with the positive semidefinite constraints folded into the domain of the loss, and Dykstra is Dykstra’s adaptation of alternating projections. All times are in seconds.

Size	PD1		PD2		PD3		Dykstra	
*n*	Loss	Time	Loss	Time	Loss	Time	Loss	Time
2	1.64	0.36	1.64	0.01	1.64	0.01	1.64	0.00
4	2.86	0.10	2.86	0.01	2.86	0.01	2.86	0.00
8	18.77	0.21	18.78	0.03	18.78	0.03	18.78	0.00
16	45.10	0.84	45.12	0.18	45.12	0.12	45.12	0.02
32	169.58	4.36	169.70	0.61	169.70	0.52	169.70	0.37
64	837.85	16.77	838.44	2.90	838.43	2.63	838.42	4.32
128	3276.41	91.94	3279.44	18.00	3279.25	14.83	3279.23	19.73
256	14029.07	403.59	14045.30	89.58	14043.59	64.89	14043.46	72.79

**Table 4:** CPU times and optima for the second-order cone projection. Here *m* is the number of constraints, *n* is the number of variables, PD is the accelerated proximal distance algorithm, SCS is the Splitting Cone Solver, and Gurobi is the Gurobi solver. After *m* = 512 the constraint matrix ***A*** is initialized with sparsity level 0.01.

Dimensions		Optima			CPU Seconds		
*m*	*n*	PD	SCS	Gurobi	PD	SCS	Gurobi
2	4	0.10598	0.10607	0.10598	0.0043	0.0103	0.0026
4	8	0.00000	0.00000	0.00000	0.0003	0.0009	0.0022
8	16	0.88988	0.88991	0.88988	0.0557	0.0011	0.0027
16	32	2.16514	2.16520	2.16514	0.0725	0.0012	0.0040
32	64	3.03855	3.03864	3.03853	0.0952	0.0019	0.0094
64	128	4.86894	4.86962	4.86895	0.1225	0.0065	0.0403
128	256	10.5863	10.5843	10.5863	0.1975	0.0810	0.0868
256	512	31.1039	31.0965	31.1039	0.5463	0.3995	0.3405
512	1024	27.0483	27.0475	27.0483	3.7667	1.6692	2.0189
1024	2048	1.45578	1.45569	1.45569	0.5352	0.3691	1.5489
2048	4096	2.22936	2.22930	2.22921	1.0845	2.4531	5.5521
4096	8192	1.72306	1.72202	1.72209	3.1404	17.272	15.204
8192	16384	5.36191	5.36116	5.36144	13.979	133.25	88.024

**Table 5:** CPU times (seconds) and optima for approximating the Horn variational index of a Horn matrix. Here *n* is the size of Horn matrix, PD is the proximal distance algorithm, aPD is the accelerated proximal distance algorithm, and Mosek is the Mosek solver.

Dimension	Optima			CPU Seconds		
*n*	PD	aPD	Mosek	PD	aPD	Mosek
4	0.000000	0.000000	feasible	0.5555	0.0124	2.7744
5	0.000000	0.000000	infeasible	0.0039	0.0086	0.0276
8	0.000021	0.000000	feasible	0.0059	0.0083	0.0050
9	0.000045	0.000000	infeasible	0.0055	0.0072	0.0082
16	0.000377	0.000001	feasible	0.0204	0.0237	0.0185
17	0.000441	0.000001	infeasible	0.0204	0.0378	0.0175
32	0.001610	0.000007	feasible	0.0288	0.0288	0.1211
33	0.002357	0.000009	infeasible	0.0242	0.0346	0.1294
64	0.054195	0.000026	feasible	0.0415	0.0494	3.6284
65	0.006985	0.000026	infeasible	0.0431	0.0551	2.7862

**Table 6:** CPU times and optima for testing the copositivity of random symmetric matrices. Here *n* is the size of matrix, PD is the proximal distance algorithm, aPD is the accelerated proximal distance algorithm, and Mosek is the Mosek solver.

Dimension	Optima			CPU Seconds		
*n*	PD	aPD	Mosek	PD	aPD	Mosek
4	−0.391552	−0.391561	infeasible	0.0029	0.0031	0.0024
8	−0.911140	−2.050316	infeasible	0.0037	0.0044	0.0045
16	−1.680697	−1.680930	infeasible	0.0199	0.0272	0.0062
32	−2.334520	−2.510781	infeasible	0.0261	0.0242	0.0441
64	−3.821927	−3.628060	infeasible	0.0393	0.0437	0.6559
128	−5.473609	−5.475879	infeasible	0.0792	0.0798	38.3919
256	−7.956365	−7.551814	infeasible	0.1632	0.1797	456.1500

**Table 7:** CPU times (seconds) and optima for the linear complementarity problem with randomly generated data. Here *n* is the size of matrix, PD is the accelerated proximal distance algorithm, and Gurobi is the Gurobi solver.

Dimension	Optima		CPU Seconds	
*n*	PD	Mosek	PD	Mosek
4	0.000000	0.000000	0.0230	0.0266
8	0.000000	0.000000	0.0062	0.0079
16	0.000000	0.000000	0.0269	0.0052
32	0.000000	0.000000	0.0996	0.4303
64	0.000074	0.000000	2.6846	360.5183

## Appendix A. Proofs of the Stated Propositions

### A.1. Proposition 1

We first observe that the surrogate function *g*_*ρ*_(*x* | *x*_*k*_) is *ρ*-strongly convex. Consequently, the stationarity condition $0\in \partial g_{\rho }\left(x_{k+1}|x_{k}\right)$ implies

(20) $$g_{\rho }\left(x|x_{k}\right)\geq g_{\rho }\left(x_{k+1}|x_{k}\right)+\frac{\rho }{2}{\left\|x-x_{k+1}\right\|}^{2}$$

for all *x*. In the notation (9), the difference

$$d_{\rho }\left(x|x_{k}\right)=g_{\rho }\left(x|x_{k}\right)-h_{\rho }\left(x\right)=\frac{\rho }{2}{\left\|x-y_{k}\right\|}^{2}-\frac{\rho }{2}\overset{m}{\underset{i=1}{\sum }}\alpha_{i}\text{dist}{\left(x,C_{i}\right)}^{2}$$

is *ρ*-smooth because

$$\nabla d_{\rho }\left(x|x_{k}\right)=\rho \left(x-y_{k}\right)-\rho \overset{m}{\underset{i=1}{\sum }}\alpha_{i}\left[x-P_{C_{i}}\left(x\right)\right]=\rho \overset{m}{\underset{i=1}{\sum }}\alpha_{i}P_{C_{i}}\left(x\right)-\rho y_{k}.$$

The tangency conditions *d*_*ρ*_(*x*_*k*_ | *x*_*k*_) = 0 and ∇*d*_*ρ*_(*x*_*k*_ | *x*_*k*_) = **0** therefore yield

(21) $$d_{\rho }\left(x|x_{k}\right)\leq d_{\rho }\left(x_{k}|x_{k}\right)+\nabla d_{\rho }{\left(x_{k}\right)}^{t}\left(x-x_{k}\right)+\frac{\rho }{2}{\left\|x-x_{k}\right\|}^{2}=\frac{\rho }{2}{\left\|x-x_{k}\right\|}^{2}$$

for all *x*. Combining inequalities (20) and (21) gives

$$h_{\rho }\left(x_{k+1}\right)+\frac{\rho }{2}\|x-x_{k+1}{\|}^{2}\leq g_{\rho }\left(x_{k+1}|x_{k}\right)+\frac{\rho }{2}\|x-x_{k+1}{\|}^{2}\;\leq g_{\rho }\left(x|x_{k}\right)\;=h_{\rho }(x)+d_{\rho }\left(x|x_{k}\right)\;\leq h_{\rho }(x)+\frac{\rho }{2}\|x-x_{k}{\|}^{2}.$$

Adding the result

$$h_{\rho }\left(x_{k+1}\right)-h_{\rho }(x)\leq \frac{\rho }{2}\left(\|x-x_{k}{\|}^{2}-\|x-x_{k+1}{\|}^{2}\right)$$

over *k* and invoking the descent property *h*_*ρ*_(***x***_*k*+1_) ≤ *h*_*ρ*_(***x***_*k*_) produce the error bound

$$h_{\rho }\left(x_{k+1}\right)-h_{\rho }(x)\leq \frac{\rho }{2(k+1)}\left(\|x-x_{0}{\|}^{2}-\|x-x_{k+1}{\|}^{2}\right)\leq \frac{\rho }{2(k+1)}\|x-x_{0}{\|}^{2}.$$

Setting *x* equal to a minimal point *z* gives the stated result. ■

### A.2. Proposition 2

The existence and uniqueness of ***y*** are obvious. The remainder of the proof hinges on the assumptions that *h*_*ρ*_(***x***) is *μ*-strongly convex and the surrogate *g*_*ρ*_(***x*** | ***x***_*k*_) is *L* + *ρ* smooth. The latter assumption yields

(22) $$h_{\rho }\left(x\right)-h_{\rho }\left(y\right)\leq g_{\rho }\left(x|y\right)-g_{\rho }\left(y|y\right)\;\leq \nabla g_{\rho }{\left(y|y\right)}^{t}\left(x-y\right)+\frac{L+\rho }{2}{\left\|x-y\right\|}^{2}\;=\frac{L+\rho }{2}{\left\|x-y\right\|}^{2}.$$

The strong convexity condition $h_{\rho }(y)-h_{\rho }(x)\geq \nabla h_{\rho }{\left(x\right)}^{t}\left(y-x\right)+\frac{\mu }{2}\|y-x{\|}^{2}$ implies

$$\|\nabla h_{\rho }(x)\|\cdot \|y-x\|\;\geq -\nabla h_{\rho }{\left(x\right)}^{t}(y-x)\geq \frac{\mu }{2}\|y-x{\|}^{2}.$$

It follows that $\left\|\nabla h_{\rho }(x)\|\geq \frac{\mu }{2}\|x-y\right\|$. This last inequality and inequality (22) produce the Polyak-Łojasiewicz bound

$$\frac{1}{2}\|\nabla h_{\rho }(x){\|}^{2}\geq \frac{\mu^{2}}{2(L+\rho )}\left[h_{\rho }(x)-h_{\rho }(y)\right].$$

Taking $x=x_{k}-\frac{1}{L+\rho }\nabla g_{\rho }\left(x_{k}|x_{k}\right)=x_{k}-\frac{1}{L+\rho }\nabla h_{\rho }\left(x_{k}\right)$, the Polyak-Łojasiewicz bound gives

$$h_{\rho }\left(x_{k+1}\right)-h_{\rho }\left(x_{k}\right)\leq g_{\rho }\left(x_{k+1}|x_{k}\right)-g_{\rho }\left(x_{k}|x_{k}\right)\;\leq g_{\rho }\left(x|x_{k}\right)-g_{\rho }\left(x_{k}|x_{k}\right)\;\leq -\frac{1}{L+\rho }\nabla g_{\rho }{\left(x_{k}|x_{k}\right)}^{t}\nabla h_{\rho }\left(x_{k}\right)+\frac{1}{2(L+\rho )}\|\nabla h_{\rho }\left(x_{k}\right){\|}^{2}\;=-\frac{1}{2(L+\rho )}\|\nabla h_{\rho }\left(x_{k}\right){\|}^{2}\;\leq -\frac{\mu^{2}}{2{\left(L+\rho \right)}^{2}}\left[h_{\rho }\left(x_{k}\right)-h_{\rho }(y)\right].$$

Rearranging this inequality yields

$$h_{\rho }\left(x_{k+1}\right)-h_{\rho }(y)\leq \left[1-\frac{\mu^{2}}{2{\left(L+\rho \right)}^{2}}\right]\left[h_{\rho }\left(x_{k}\right)-h_{\rho }(y)\right],$$

which can be iterated to give the stated bound. ■

### A.3. Proposition 3

Consider first the proximal distance iterates. The inequality

$$f\left(x_{k}\right)+\frac{\rho_{k}}{2}\text{dist}{\left(x_{k},S\right)}^{2}\leq f\left(x_{k}\right)+\frac{\rho_{k}}{2}\|x_{k}-P_{S}\left(x_{k-1}\right){\|}^{2}\leq f\left[P_{S}\left(x_{k-1}\right)\right]\leq \underset{x\in S}{\text{sup}}f(x)$$

plus the coerciveness of *f*(***x***) imply that ***x***_*k*_ is a bounded sequence. The claimed bound now holds for *c* equal to the finite supremum of the sequence 2[sup_*x*∈*S*_ *f*(***x***) − *f*(***x***_*k*_)]. If in addition, *f*(***x***) is continuously differentiable, then the stationarity equation

$$0=\nabla f\left(x_{k}\right)+\rho_{k}\left[x_{k}-P_{S}\left(x_{k-1}\right)\right]$$

and the Cauchy-Schwarz inequality give

$$\rho_{k}\|x_{k}-P_{S}\left(x_{k-1}\right){\|}^{2}=-\nabla f{\left(x_{k}\right)}^{t}\left[x_{k}-P_{S}\left(x_{k-1}\right)\right]\leq \|\nabla f\left(x_{k}\right)\|\cdot \|x_{k}-P_{S}\left(x_{k-1}\right)\|.$$

Dividing this by ‖*x*_*k*_ − *P*_*S*_(*x*_*k*−1_) ‖ and squaring further yield

$$\rho_{k}^{2}\text{dist}{\left(x_{k},S\right)}^{2}\leq \rho_{k}^{2}\|x_{k}-P_{S}\left(x_{k-1}\right){\|}^{2}\leq \|\nabla f\left(x_{k}\right){\|}^{2}.$$

Taking *d* = sup_*k*_ ‖∇*f*(*x*_*k*_) ‖^2^ over the bounded sequence ***x***_*k*_ completes the proof. For the penalty method iterates, the bound

$$f\left(y_{k}\right)+\frac{\rho_{k}}{2}\text{dist}{\left(y_{k},S\right)}^{2}\leq f(y)$$

is valid by definition, where ***y*** attains the constrained minimum. Thus, coerciveness implies that the sequence ***y***_*k*_ is bounded. When *f*(***x***) is continuously differentiable, the proof of the second claim also applies if we substitute ***y***_*k*_ for ***x***_*k*_ and *P*_*S*_(***y***_*k*_) for *P*_*S*_(***x***_*k*−1_). ■

### A.4. Proposition 4

Because the function $f(x)+\frac{\rho_{k}}{2}\text{dist}{\left(x,S\right)}^{2}$ is convex and has the value *f*(***y***) and gradient ∇*f*(***y***) at a constrained minimum ***y***, the supporting hyperplane principle says

$$f\left(y_{k}\right)+\frac{\rho_{k}}{2}\text{dist}{\left(y_{k},S\right)}^{2}\geq f(y)+\nabla f{\left(y\right)}^{t}\left(y_{k}-y\right)\;=f(y)+\nabla f{\left(y\right)}^{t}\left[P_{S}\left(y_{k}\right)-y\right]+\nabla f{\left(y\right)}^{t}\left[y_{k}-P_{S}\left(y_{k}\right)\right].$$

The first-order optimality condition ∇*f*(***y***)^*t*^[*P*_*S*_(***y***_*k*_) − ***y***] ≥ 0 holds given ***y*** is a constrained minimum. Hence, the Cauchy-Schwarz inequality and Proposition 3 imply

$$f(y)-f\left(y_{k}\right)\leq \frac{\rho_{k}}{2}\text{dist}{\left(y_{k},S\right)}^{2}-\nabla f{\left(y\right)}^{t}\left[y_{k}-P_{S}\left(y_{k}\right)\right]\;\leq \frac{\rho_{k}}{2}\text{dist}{\left(y_{k},S\right)}^{2}+\|\nabla f(y)\|\cdot \text{dist}\left(y_{k},S\right)\;\leq \frac{d+2\sqrt{d}\|\nabla f(y)\|}{2\rho_{k}}.$$

■

### A.5. Proposition 5

Let ***x***_*k*_ converge to ***x***_∞_ and ***y***_*k*_ ∈ *P*_*S*_(***x***_*k*_) converge to ***y***_∞_. For an arbitrary ***y*** ∈ *S*, taking limits in the inequality ‖***x***_*k*_ −***y***_*k*_‖ ≤ ‖***x***_*k*_–***y***‖ yields ‖***x***_∞_ −***y***_∞_‖ ≤ ‖*x*_∞_ −*y*‖; consequently, ***y***_∞_ ∈ *P*_*S*_(***x***_∞_). To prove the second assertion, take ***y***_*k*_∈ *P*_*S*_(***x***_*k*_) and observe that

$$\|y_{k}\|\leq \|x_{k}-y_{k}\|+\|x_{k}\|\;\leq \|x_{k}-y_{1}\|+\|x_{k}\|\;\leq \|x_{k}-x_{1}\|+\|x_{1}-y_{1}\|+\|x_{k}\|\;\leq \|x_{k}\|+\|x_{1}\|+\text{dist}\left(x_{1},S\right)+\|x_{k}\|,$$

which is bounded above by the constant dist(***x***_1_*,S*) + 3^sup^_*m*≥1_ ‖*x*_*m*_‖. ■

### A.6. Proposition 6

In fact, a much stronger result holds. Since the function *s*(***x***) of equation (8) is convex and finite, Alexandrov’s theorem (Niculescu and Persson, 2006) implies that it is almost everywhere twice differentiable. In view of the identities $\frac{1}{2}\text{dist}{\left(x,S\right)}^{2}=\frac{1}{2}\|x{\|}^{2}-s(x)$ and $x-P_{S}(x)=\nabla \frac{1}{2}\text{dist}{\left(x,S\right)}^{2}$ where *P*_*S*_(***x***) is single valued, it follows that *P*_*S*_(***x***) = ∇*s*(***x***) is almost everywhere differentiable. ■

### A.7. Proposition 8

See the discussion just prior to the statement of the proposition. ■

### A.8. Proposition 9

The strong-convexity inequality

$$g_{\rho }\left(x_{k}|x_{k}\right)\geq g_{\rho }\left(x_{k+1}|x_{k}\right)+\frac{\mu }{2}\|x_{k}-x_{k+1}{\|}^{2}$$

and the tangency and domination properties of the algorithm imply

(23) $$h_{\rho }\left(x_{k}\right)-h_{\rho }\left(x_{k+1}\right)\geq \frac{\mu }{2}\|x_{k}-x_{k+1}{\|}^{2}.$$

Since the difference in function values tends to 0, this validates the stated limit. The remaining assertions follow from Propositions 7.3.3 and 7.3.5 of (Lange, 2016). ■

### A.9. Proposition 10

Let the subsequence $x_{k_{m}}$ of the MM sequence *x*_*k*+1_ ∈ *M*(***x***_*k*_) converge to *z*. By passing to a subsubsequence if necessary, we may suppose that ***x***_*km*+1_ converges to ***y***. Owing to our closedness assumption, ***y*** ∈ *M*(*z*). Given that *h*(***y***) = *h*(*z*), it is obvious that *z* also minimizes *g*(***x*** | *z*) and that **0** = ∇*g*(*z* | *z*). Since the difference Δ(*x* | *z*) = *g*(*x* | *z*) − *h*(*x*) achieves its minimum at *x* = *z*, the Fréchet subdifferential *∂*^*F*^ Δ(*x* | *z*) satisfies

$$0\in \partial^{F}\Delta \left(z|z\right)=\nabla g\left(z|z\right)+\partial^{F}(-h)(z).$$

It follows that **0** ∈ *∂*^*F*^ (−*h*)(***z***). ■

### A.10. Proposition 11

Because Δ(***x*** | ***y***) = *g*(***x*** | ***y***) − *h*(***x***) achieves its minimum at ***x*** = ***y***, the Fréchet subdifferential *∂*^*F*^ Δ(***x*** | ***y***) satisfies

$$0\in \partial^{F}\Delta \left(y|y\right)=\nabla g\left(y|y\right)+\partial^{F}(-h)(y).$$

It follows that −∇*g*(***y*** | ***y***) ∈ *∂*^*F*^ (−*h*)(***y***). Furthermore, by assumption

$$\left\|\nabla g\left(a|x_{k}\right)-\nabla g\left(b|x_{k}\right)\|\leq L\|a-b\right\|$$

for all relevant ***a*** and ***b*** and ***x***_*k*_. In particular, because ∇*g*(***x***_*k*+1_ | ***x***_*k*_) = **0**, we have

(24) $$\|\nabla g\left(x_{k}|x_{k}\right)\|\leq L\|x_{k+1}-x_{k}\|.$$

Let *W* denote the set of limit points. The objective *h*(***x***) is constant on *W* with value $\bar{h}={\text{lim}}_{k\to \infty }h\left(x_{k}\right)$. According to the Łojasiewicz inequality applied for the subanalytic function $\bar{h}-h(x)$, for each ***z*** ∈ *W* there exists an open ball *B*_*r*(*z*)_(*z*) of radius *r*(*z*) around ***z*** and an exponent *θ*(***z***) ∈ [0,1) such that

$${\left|h(u)-h(z)\right|}^{\theta (z)}={\left|\bar{h}-h(u)-\bar{h}+\bar{h}\right|}^{\theta (z)}\leq c(z)\|v\|$$

for all ***u*** ∈ *B*_*r*(*z*)_(*z*) and all $v\in \partial^{F}(\bar{h}-h)(u)=\partial^{F}(-h)(u)$. We will apply this inequality to ***u*** = ***x***_*k*_ and ***v*** = −∇*g*(***x***_*k*_ | ***x***_*k*_). In so doing, we would like to assume that the exponent *θ*(*z*) and constant *c*(***z***) do not depend on ***z***. With this end in mind, cover the compact set *W* by a finite number of balls *B*_*r*(*zi*_)(*z*_*i*_) and take *θ* = max_*i*_ *θ*(*z*_*i*_) < 1 and *c* = max_*i*_ *c*(***z***_*i*_). For a sufficiently large *K*, every ***x***_*k*_ with *k* ≥ *K* falls within one of these balls and satisfies $\left|\bar{h}-h\left(x_{k}\right)\right|<1$. Without loss of generality assume *K* = 0. The Łojasiewicz inequality reads

(25) $${\left|\bar{h}-h\left(x_{k}\right)\right|}^{\theta }\leq c\|\nabla g\left(x_{k}|x_{k}\right)\|.$$

In combination with the concavity of the function *t*^1−*θ*^ on [0,∞), inequalities (23), (24), and (25) imply

$${\left[h\left(x_{k}\right)-\bar{h}\right]}^{1-\theta }-{\left[h\left(x_{k+1}\right)-\bar{h}\right]}^{1-\theta }\geq \frac{1-\theta }{{\left[h\left(x_{k}\right)-\bar{h}\right]}^{\theta }}\left[h\left(x_{k}\right)-h\left(x_{k+1}\right)\right]\;\geq \frac{1-\theta }{c\|\nabla g\left(x_{k}|x_{k}\right)\|}\frac{\mu }{2}\|x_{k+1}-x_{k}{\|}^{2}\;\geq \frac{(1-\theta )\mu }{2cL}\|x_{k+1}-x_{k}\|.$$

Rearranging this inequality and summing over *k* yield

$$\overset{\infty }{\underset{n=0}{\sum }}\|x_{k+1}-x_{k}\|\leq \frac{2cL}{(1-\theta )\mu }{\left[h\left(x_{0}\right)-\bar{h}\right]}^{1-\theta }$$

Thus, the sequence ***x***_*k*_ is a fast Cauchy sequence and converges to a unique limit in *W*. ■
