# Supplementary figures and images for: Proximal Distance Algorithms: Theory and Practice
Source: J Mach Learn Res. Author manuscript; Available in PMC 2019 Oct 24. (PMC6812563)

Compute time to calculate q Sparse PCs

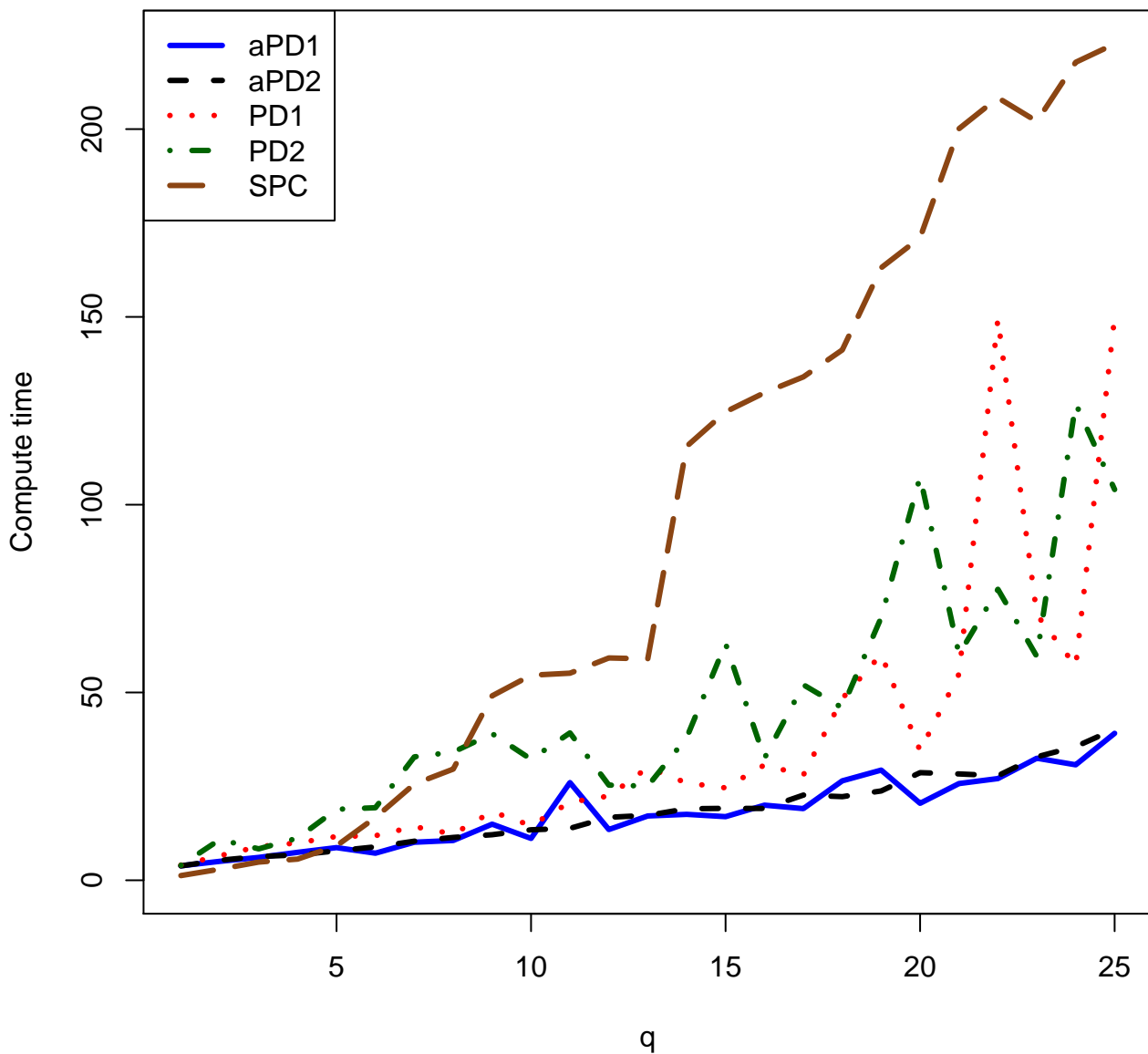

Supplement: code [file NIHMS1053815-supplement-code.zip › proxdist-master/keys_zhou_lange_2019/misc/spca_results_k25_sumabsv8_noaccel_time.pdf]

PVE of q Sparse PCs

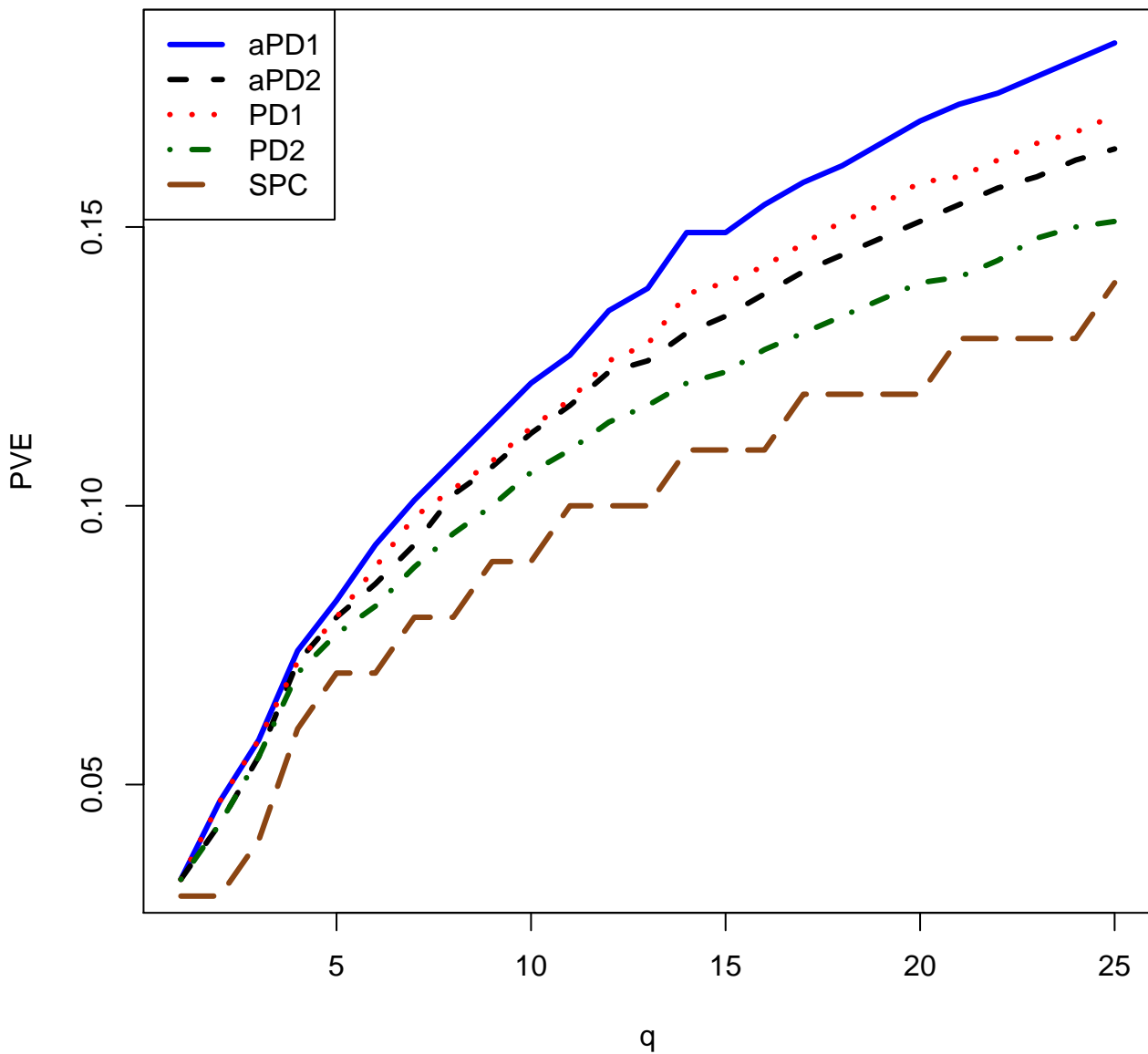

Supplement: code [file NIHMS1053815-supplement-code.zip › proxdist-master/keys_zhou_lange_2019/misc/spca_results_k25_sumabsv8_noaccel_pve.pdf]
