# Supplementary material for: Proximal Distance Algorithms: Theory and Practice
Source: J Mach Learn Res. Author manuscript; Available in PMC 2019 Oct 24. (PMC6812563)
Supplement: code [file NIHMS1053815-supplement-code.zip › proxdist-master/keys_zhou_lange_2019/spca/spca_results_k25_sumabsv8_pve.pdf]

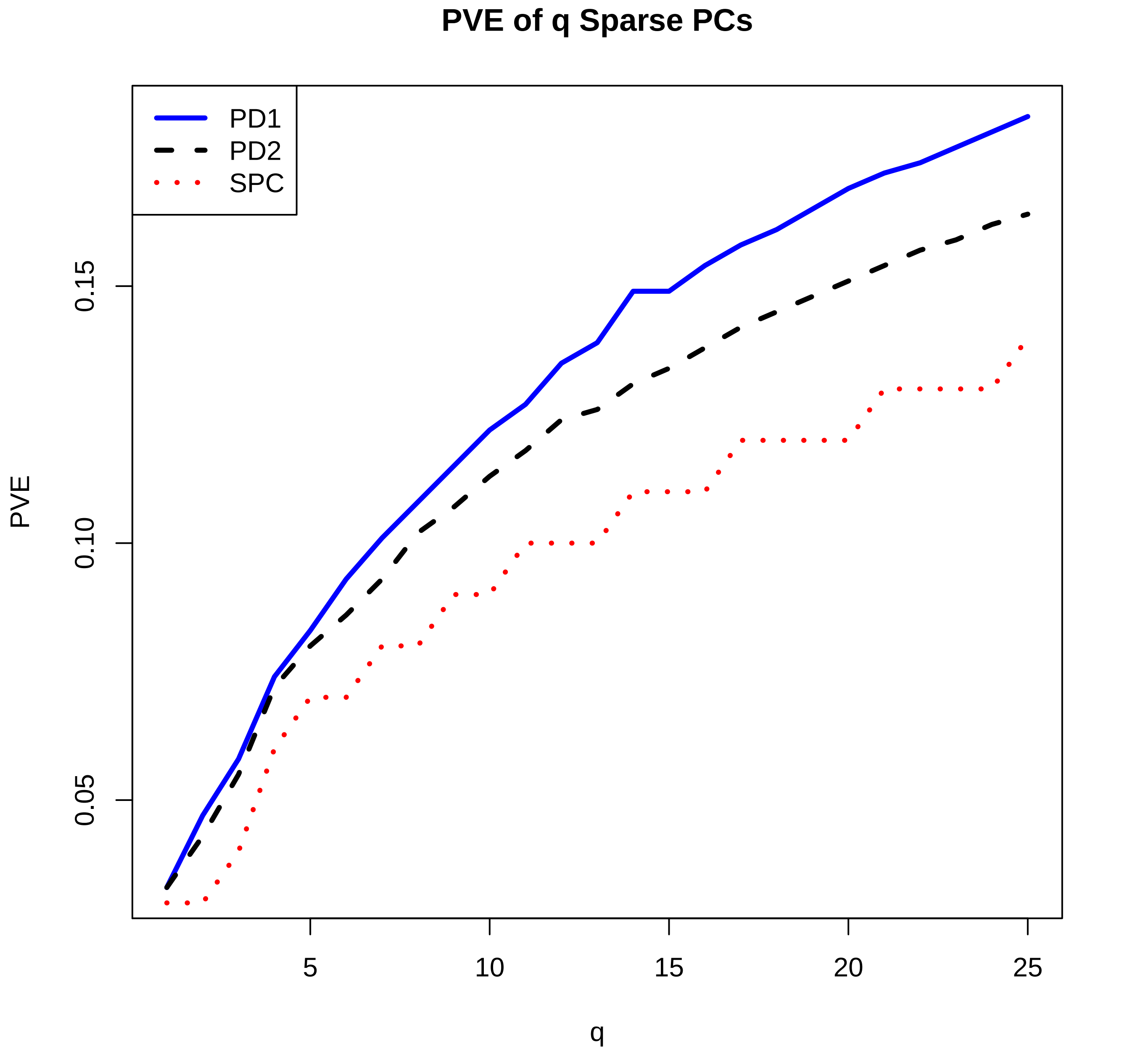
PVE of q Sparse PCs
PD1
PD2
SPC
PVE
0.05
0.10
0.15
q
5
10
15
20
25
