# Supplementary material for: Proximal Distance Algorithms: Theory and Practice
Source: J Mach Learn Res. Author manuscript; Available in PMC 2019 Oct 24. (PMC6812563)
Supplement: code [file NIHMS1053815-supplement-code.zip › proxdist-master/keys_zhou_lange_2019/spca/spca_results_k25_sumabsv8_time.pdf]

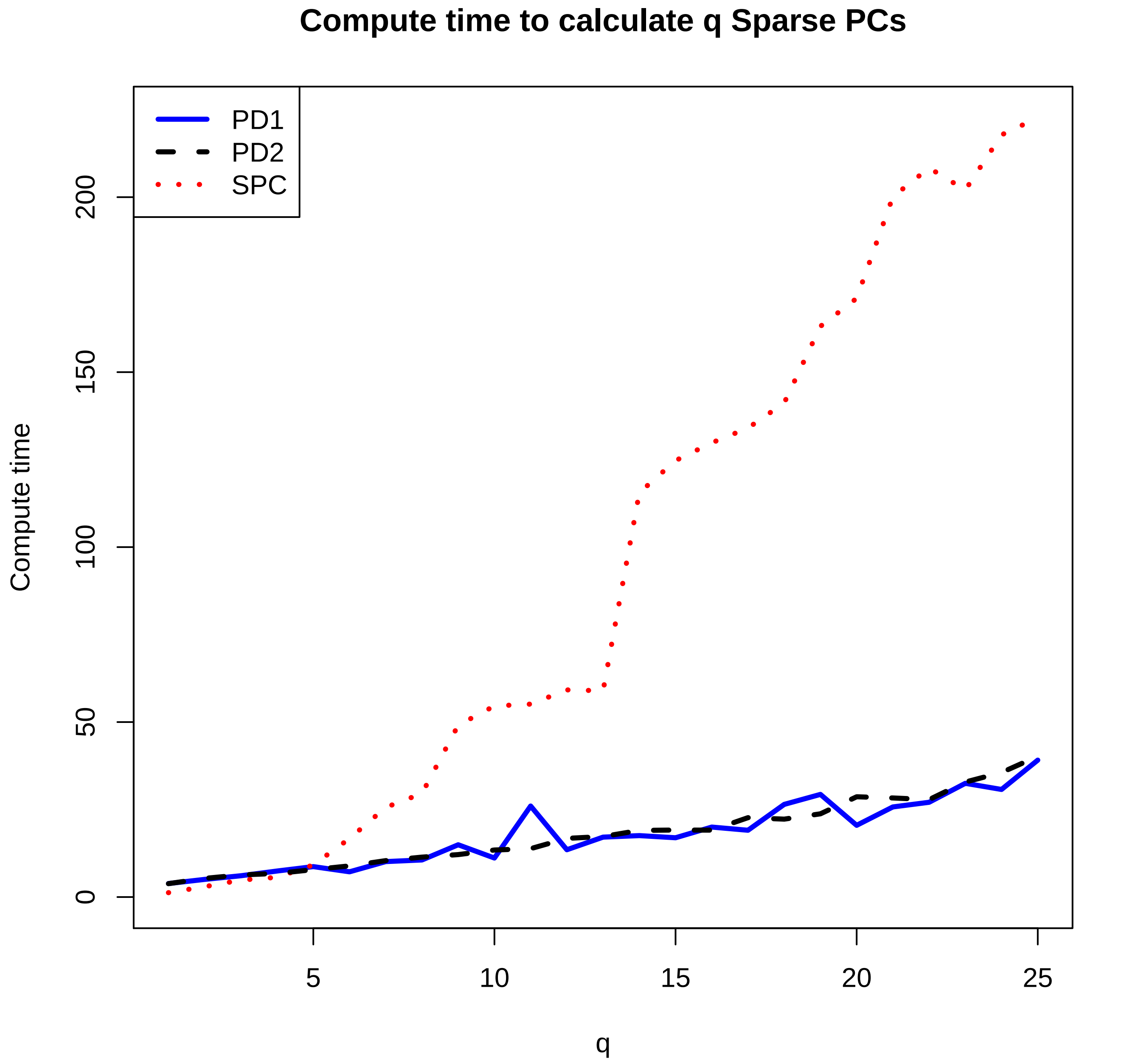
Compute time to calculate q Sparse PCs
PD1
PD2
SPC
Compute time
0
50
100
150
200
q
5
10
15
20
25
